# Steric hindrance modulation of hexaazatribenzanthraquinone isomers for high-capacity and wide-temperature-range aqueous proton battery

**DOI:** 10.1093/nsr/nwae045

**Published:** 2024-02-01

**Authors:** Mingsheng Yang, Yuxin Hao, Bei Wang, Yan Wang, Liping Zheng, Rui Li, Huige Ma, Xinyu Wang, Xiaoming Jing, Hongwei Li, Mengxiao Li, Zhihui Wang, Yujie Dai, Guangcun Shan, Mingjun Hu, Jun Luo, Jun Yang

**Affiliations:** School of Materials Science and Engineering, Beihang University, Beijing 100191, China; Beijing Institute of Nanoenergy & Nanosystems, Chinese Academy of Sciences, Beijing 101400, China; Beijing Institute of Nanoenergy & Nanosystems, Chinese Academy of Sciences, Beijing 101400, China; Center on Nanoenergy Research, School of Physical Science & Technology, Guangxi University, Nanning 530004, China; School of Chemistry and Chemical Engineering, Center on Nanoenergy Research, Guangxi University, Nanning 530004, China; Beijing Institute of Nanoenergy & Nanosystems, Chinese Academy of Sciences, Beijing 101400, China; Beijing Institute of Nanoenergy & Nanosystems, Chinese Academy of Sciences, Beijing 101400, China; Beijing Institute of Nanoenergy & Nanosystems, Chinese Academy of Sciences, Beijing 101400, China; Beijing Institute of Nanoenergy & Nanosystems, Chinese Academy of Sciences, Beijing 101400, China; School of Materials Science and Engineering, Beihang University, Beijing 100191, China; School of Materials Science and Engineering, Beihang University, Beijing 100191, China; School of Materials Science and Engineering, Beihang University, Beijing 100191, China; Beijing Institute of Nanoenergy & Nanosystems, Chinese Academy of Sciences, Beijing 101400, China; School of Instrumentation Science and Opto-electronics Engineering, Beihang University, Beijing 100191, China; School of Materials Science and Engineering, Beihang University, Beijing 100191, China; ShenSi Lab, Shenzhen Institute for Advanced Study, University of Electronic Science and Technology of China, Shenzhen 518110, China; Beijing Institute of Nanoenergy & Nanosystems, Chinese Academy of Sciences, Beijing 101400, China; ShenSi Lab, Shenzhen Institute for Advanced Study, University of Electronic Science and Technology of China, Shenzhen 518110, China

**Keywords:** stereoisomers, steric effect, aqueous battery, super cycling stability, ultra-low temperature proton battery

## Abstract

Organic materials with rich active sites are good candidates of high-capacity anodes in aqueous batteries, but commonly low utilization of active sites limits their capacity. Herein, two isomers, symmetric and asymmetric hexaazatribenzanthraquinone (s-HATBAQ and a-HATBAQ), with rich active sites have been synthesized in a controllable manner. It has been revealed for the first time that a sulfuric acid catalyst can facilitate the stereoselective formation of s-HATBAQ. Attributed to the reduced steric hindrance in favor of proton insertion as well as the amorphous structure conducive to electrochemical dynamics, s-HATBAQ exhibits 1.5 times larger specific capacity than a-HATBAQ. Consequently, the electrode of s-HATBAQ with 50% reduced graphene oxide (s-HATBAQ-50%rGO) delivers a record high specific capacity of 405 mAh g^−1^ in H_2_SO_4_ electrolyte. Moreover, the assembled MnO_2_//s-HATBAQ-50%rGO aqueous proton full batteries show an exceptional cycling stability at 25°C and can maintain ∼92% capacity after 1000 cycles at 0.5 A g^−1^ at −80°C. This work demonstrates the controllable synthesis of isomers, showcases a wide-temperature-range prototype proton battery and highlights the significance of precise molecular structure modulation in organic energy storage.

## Teaser

This paper describes the controllable synthesis of two isomeric conjugated small molecules in an archiral environment and their wide-temperature-range proton storage behaviors, and confirms the important role of steric hindrance in determining small-molecule electrochemical reactivity.

## INTRODUCTION

Aqueous batteries (ABs) that employ water as the electrolyte solvent fundamentally solve the intrinsic challenges caused by flammable organic electrolytes, such as safety concerns, strict manufacturing conditions, and the cost of expensive electrolytes [1,2]. Among ABs, aqueous proton batteries (APBs) have been regarded as one of the most promising energy storage devices because of the small size, ultrafast diffusion kinetics, and wide availability of protons [3–10]. The first rechargeable device using protons as charge carriers can date back to lead acid batteries in 1859 [11,12]. However, the low specific power density, unsatisfactory cycle life, energy efficiency, and lead toxicity considerably limited the application range of these batteries in low-speed electric vehicles and uninterruptible power supplies (UPS) [13]. Recently, different genres of electrode materials have been explored for proton storage [14–23]. Among them, electrodeposited MnO_2_ was widely used as the cathode of APBs, due to its high discharge voltage (1.22 V vs. SHE) and high specific capacity (616 mAh g^−1^) [23,24]. With the attributes of high redox reversibility, good sustainability, strong acid resistance, and cost effectiveness, organic compounds have been the preferred choice of anode materials [25–27]. However, despite high theoretical specific capacity, the low real capacity of organic electrodes, in most cases resulting from low utilization of active sites, still largely limits their actual application.

It has been well-known that the electrochemical properties of organic electrodes are greatly affected by the electronic effects of their molecular structure, mainly including inductive effects, conjugated effects, and electric field effects. For example, the voltage of organic electrode materials can be effectively adjusted by introducing electron-withdrawing/donating groups into the molecular skeletons due to their inductive electronic effect, which can regulate the LUMO and HOMO energy levels [28]. Conjugated structure and conjugated effects also play critical roles in determining charge transport and the reversibility of redox reactions [29]. In addition, field effect can exert obvious influence on charge transport and occupation, thus affecting the capacity and rate performance. Different from the electronic effects, the steric effect works by controlling the accessibility of active sites based on the physical blocking to insertion ions. Recently, there have been several reports that describe the influence of the steric effect on the electrochemical properties of organic electrode materials [28,30–35]. However, in the reported works, the controlled synthesis of stereoisomers in an achiral environment and the influence of their precise structures on electrochemical behaviors have rarely been mentioned, but actually it is of high value in both science and engineering to investigate the relationship between the steric hindrance of the stereoisomers and the electrochemical performance for guiding the precise structure design of organic electrodes and excavating their full potential in energy storage.

In the past few years, hexaazatrinaphthalene (HATN)-based organic molecules have been a class of star electrode materials in energy storage, due to their high specific capacity and good cycling stability [36–39]. Most reported HATN-based organic electrode materials exhibited highly symmetric molecular structures, conferring them with uniform and reliable electrochemical properties. Recently, a novel HATN-based molecule, hexaazatribenzanthraquinone (HATBAQ), had been synthesized and used as the electrodes of a supercapacitor, with its actual capacity (43.1 F g^−1^ or 9.5 mAh g^−1^ at 0.5 A g^−1^ in 1 M H_2_SO_4_) being lower than the expected capacity (415 mAh g^−1^) [40]. This significant result indicates that HATBAQ owns two stereoisomers, the symmetric one (s-HATBAQ) and the asymmetric one (a-HATBAQ), which means that their different molecular structures may cause different electrochemical properties, and their random mixing may be the reason for the lower capacity. Thus, the precise modulation between the a-HATBAQ and the s-HATBAQ molecular structures is indispensable in promoting the electrochemical properties of HATBAQ, and this strategy is also important for other organic electrode materials. Additionally, it is very meaningful to explore the controllable synthesis of isomers and find the leading factors that result in their selective formation.

**Figure 1.**
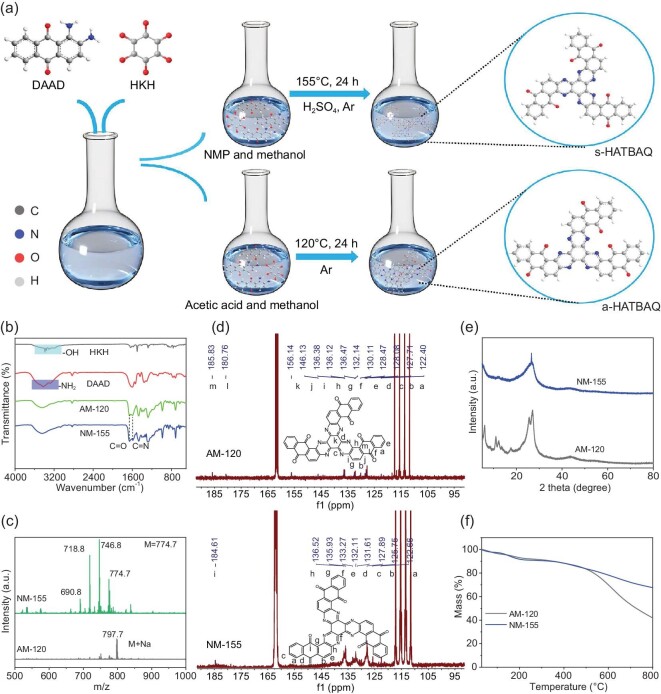
(a) Schematic illustration of the synthetic route for s-HATBAQ and a-HATBAQ. (b) FT-IR spectra of AM-120 and NM-155. (c) Mass spectra of AM-120 and NM-155. (d) ^13^C NMR spectra of AM-120 and NM-155. (e) XRD patterns of AM-120 and NM-155. (f) TGA curves of AM-120 and NM-175.

To identify the above conjecture, herein, we synthesize the two isomers of s-HATBAQ and a-HATBAQ, whose molecular structures are shown in Fig. 1a, and investigate their electrochemical performance. It is the first time to report that a sulfuric acid catalyst plays a critical role in controlling the synthesis of HATBAQ isomers, and fast reversible chemical reaction dynamics in strong acid favors the formation of the thermodynamic product s-HATBAQ. Density functional theory (DFT) reveals that s-HATBAQ can combine with 12 H^+^ due to favorable steric effects, while a-HATBAQ can only combine with 11 H^+^, suggesting higher utilization of active sites for s-HATBAQ. Additionally, the experimental results demonstrate that a-HATBAQ shows better crystallinity than s-HATBAQ which may be attributed to stronger polarization of a-HATBAQ molecules. It is interesting that, compared with crystal a-HATBAQ, amorphous s-HATBAQ structure owns smaller electrochemical impedance and faster H^+^ diffusion rate. Consequently, s-HATBAQ shows a much larger discharge capacity (202 mAh g^−1^ at 1 A g^−1^) and better rate performance (154.2 mAh g^−1^at 60 A g^−1^) than a-HATBAQ (131.5 mAh g^−1^ at 1 A g^−1^, 108.5 mAh g^−1^at 60 A g^−1^), confirming an effect of precise molecular structure and intermolecular stacking mode on electrochemical performance. To further enhance the utilization of active sites, s-HATBAQ is *in-situ* synthesized on reduced graphene oxide (rGO) for integrating the advantages of s-HATBAQ with rich active sites and carbon matrix with high conductivity. Consequently, full APBs composed of an MnO_2_ cathode, which is MnO_2_  *in-situ* electrodeposited on KOH-treated carbon felt and thus also named MnO_2_@CF-KOH, and an s-HATBAQ-50% rGO anode deliver an excellent rate performance (210 mAh g^−1^ at 2 A g^−1^ and 111 mAh g^−1^ at 80 A g^−1^) and outstanding cycling performance (capacity retention of 96% after 26 000 cycles at 5 A g^−1^) at room temperature. To prompt the operation of APBs at ultralow temperatures, an anti-freezing electrolyte (5 M H_2_SO_4_ + 0.5 M Mn (BF_4_)_2_) has been developed and exhibits a high ionic conductivity of 213.8 mS cm^−1^ at −80°C. Such an anti-freezing electrolyte endows the MnO_2_@CF-KOH//s-HATBAQ-50%rGO APB with high low-temperature tolerance and stable cycling performance at −80°C (91.9 mA h g^−1^ at 0.1 A g^−1^, 92% retention after 1000 cycles at 0.5 A g^−1^). This work will enlighten the selective synthesis of isomers from a new perspective, inspire the study on the precise molecular structure regulation of organic electrodes and prompt the development of wide-temperature-range proton batteries.

## RESULTS AND DISCUSSION

### Synthesis and characterization of s-HATBAQ and a-HATBAQ

As shown in Fig. 1a, it was assumed that isomers could be synthesized in different solvents by a keto-amine condensation reaction between hexaketocyclohexane (HKH) and 1,2-diamino-9,10-anthracenedione (DAAD). To explore the optimal reaction conditions, s-HATBAQ was synthesized in a mixed solvent of *N*-methylphthalimide (NMP) and methanol with sulfuric acid as the catalyst at 115, 135, 155, and 175°C, denoted as NM-115, NM-135, NM-155, and NM-175, respectively, as shown in Scheme S1. a-HATBAQ was synthesized in a mixed solvent of acetic acid and methanol at 120°C, denoted as AM-120, as shown in Scheme S2. According to the FT-IR spectra (Fig. 1b and Fig. S1a), the characteristic stretching vibrations of pyrazine rings (*υ*_C=N_, 1588 cm^−1^) emerge in the five products, indicating the transformation from the C=O group to the C=N linkage [31,36]. Moreover, as depicted in Fig. 1c and Fig. S1b, the MALDI-TOF-MS confirms the presence of substances with the same molecular weight as the target molecules (M = 774.71). (For NM-155, the m/z values of 746.8, 718.8, and 690.8 may be attributed to the loss of [C_2_H_4_]^+^, 2 [C_2_H_4_]^+^, and 3 [C_2_H_4_]^+^ from HATBAQ; for AM-120, the m/z value of 797.7 may be attributed to the formula [M + Na^+^]). ^13^C NMR and ^1^H NMR were used to determine the chemical structure of HATBAQ isomers. As shown in Fig. 1d, the ^13^C NMR shows that the AM-120 has 13 peaks, which matches with the 13 types of C atoms in the a-HATBAQ, confirming the successful synthesis of a-HATBAQ in a mixture of methanol and acetic acid. The ^13^C NMR shows that the NM-155 has nine peaks, which matches with the nine types of C atoms in the s-HATBAQ, confirming the successful synthesis of s-HATBAQ in a mixture of methanol and NMP (Fig. S2 and Fig. 1d). These obvious four peaks of 105–120 ppm were mainly ascribed to the residual solvent CF_3_CO_2_-D. ^1^H NMR spectra further prove the successful synthesis of a-HATBAQ and s-HATBAQ (Fig. S3).

X-ray diffraction (XRD) and thermal gravimetric analysis (TGA) were further conducted to evaluate the crystallinity and thermal stability of the isomers, respectively. As shown in Fig. 1e, AM-120 presents obvious XRD diffraction peaks, suggesting a good crystallinity. However, NM-115, NM-135, NM-155 and NM-175 show an amorphous structure (Fig. S4a), which may be beneficial for the ion transport and reaction kinetics of electrode materials [41]. Furthermore, the TGA curves indicate that NM-155 have better thermal stability than AM-120 (Fig. 1f and Fig. S4b). Scanning electron microscope (SEM) and transmission electron microscope (TEM) characterizations were carried out to explore the morphology of the obtained products. As shown in Fig. S5, AM-120 exhibits a uniform plate-like morphology derived from self-assembly of particles. In contrast, NM-155 show random particles. TEM images also present the same results (Fig. S6). X-ray photoelectron spectroscopy (XPS) was adopted to analyze the surface composition and valence state of NM-155 and AM-120 in Fig. S7. The high-resolution C1s spectrum of NM-155 (AM-120) can be deconvoluted into three peaks corresponding to the C=O at 287.0 (286.9) eV, C=N at 285.7 (285.8) eV, and C-C at 284.4 (284.7) eV (Fig. S7a and S7c) [42]. The high-resolution N1s spectrum of NM-155 (AM-120) can be deconvoluted into three peaks corresponding to NH-C at 401.4 (400.9) eV, the pyrazine-like N=C signals at 399.4 (399.5) eV, and the residual -NH_2_ groups at 398.9 (398.3) eV (Fig. S7b and S7d) [43], suggesting that the formation of pyrazine structures as a result of a ketone-amine condensation reaction. In subsequent studies, NM-155 and AM-120 are denoted as s-HATBAQ and a-HATBAQ, respectively.

To identify the main factor that induced the formation of isomers, a small amount of sulfuric acid catalyst was added to the acetic acid and methanol reaction system that was employed for the synthesis of a-HATBAQ. It was found that the original crystalline a-HATBAQ was converted into an amorphous structure (Fig. S8a), and TGA curves showed thermal stability of the products was greatly enhanced and well agreed with s-HATBAQ (Fig. S8b), implying s-HATBAQ was synthesized after adding sulfuric acid catalyst, which demonstrated the important role of sulfuric acid in controlling the molecular structure and crystalline forms of HATBAQ. It was thought that the addition of sulfuric acid catalyst can accelerate the dynamics of reversible ketone-amine condensation reactions and facilitate the structure rearrangement of HATBAQ, thus resulting in the formation of thermally stable s-HATBAQ.

### Electrochemical performance of a-HATBAQ and s-HATBAQ

**Figure 2.**
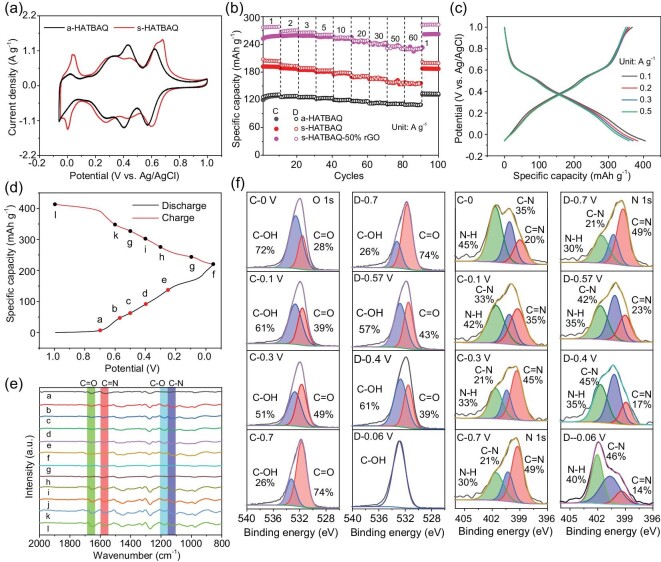
(a) CV curves and (b) rate performance of a-HATBAQ s-HATBAQ and s-HATBAQ-50% rGO. (c) GCD curves of s-HATBAQ-50% rGOs at room temperature. (d and e) *Ex-situ* FTIR and (f) O1s and N1s XPS spectra of s-HATBAQ during cycling.

HATBAQ with rich redox active sites has a high theoretical specific capacity of 415 mAh g^−1^ and is considered to be a promising anode material of APBs. It is supposed that s-HATBAQ will possess higher specific capacity than a-HATBAQ due to lower steric hindrance. To verify this above point, the electrochemical performance of a-HATBAQ and s-HATBAQ was investigated and the results were shown in Fig. 2 and Figs S9–S11. As shown in Fig. S9a and S9b, a-HATBAQ can only display three pairs of redox peaks at −0.1–1 V. Surprisingly, s-HATBAQ exhibits four pairs of redox peaks at the same voltage range (Fig. S9e), which can be ascribed to lower steric hindrance. The rate capabilities and galvanostatic charge-discharge (GCD) curves of a-HATBAQ and s-HATBAQ were then explored in the range of 1–60 A g^−1^ (Fig. 2b, Figs S10 and S11). At the current density of 1 A g^−1^, s-HATBAQ has a specific capacity of 202 mAh g^−1^ (Fig. S11c), higher than that of a-HATBAQ (131.5 mAh g^−1^), corresponding to only 49% of its theoretical capacity. This result shows that the crystallinity and molecular structure of organic electrodes can significantly affect its electrochemical properties. To understand the effect of crystallinity and molecular structure of organic electrodes on its electrochemical properties, some related experiments were conducted. As shown in Fig. S12, the porosity of a-HATBAQ and s-HATBAQ were investigated by N_2_ adsorption-desorption measurements. Although a-HATBAQ has a higher Brunauer-Emmett-Teller (BET) surface area (S_BET_) (82.074 m^2^g^−1^) than s-HATBAQ (45.712 m^2^ g^−1^) in Fig. S12a, s-HATBAQ presents a wider pore size distribution over a-HATBAQ, which is beneficial for fast transport of ions (Fig. S12b and S12c). To investigate the ion storage kinetics of a-HATBAQ and s-HATBAQ, CV measurements were carried out with scan rates ranging from 0.2 to 1 mV s^−1^ in Fig. S13. For a-HATBAQ, the b values of the three pairs of oxidation/reduction peaks were calculated to be 0.725/0.737 (O_1_/R_1_), 0.768/0.742 (O_2_/R_2_) and 0.745/0.699 (O_3_/R_3_), respectively, demonstrating the capacitive controlled process (Fig. S13a and S13b), and the b values of the four pairs of oxidation/reduction peaks from s-HATBAQ were calculated to be 0.834/0.982 (O_1_/R_1_), 0.991/0.975 (O_2_/R_2_), 0.994/0.969 (O_3_/R_3_), and 0.912 and 0.769 (O_4_/R_4_), respectively, meaning that the redox reaction dynamic is primarily dominated by a fast pseudocapacitive process (Fig. S13c and S13d). Moreover, s-HATBAQ-based cells possess lower electrochemical impedance than the a-HATBAQ-based cell (Fig. S14), suggesting faster ion transport and charge transfer for s-HATBAQ. The electrochemical kinetics and diffusion of H^+^ in a-HATBAQ and s-HATBAQ-based cells were further studied by the galvanostatic intermittent titration technique (GITT). According to the results (Fig. S15), the ${{D}_{{{H}^ + }}}$ of s-HATBAQ ranges from 1.5 × 10^−8^ to 3.6 × 10^−8^ cm^2^ s^−1^, and the ${{D}_{{{H}^ + }}}$ value of a-HATBAQ is smaller than that of s-HATBAQ ranging from 1.1 × 10^−9^ to 7.5 × 10^−9^ cm^2^ s^−1^. Therefore, it can be concluded that the electrochemical performance of HATBAQ was dictated by both molecular steric hindrance and the stacking mode (crystal or amorphous), wherein molecular steric effects should be a more essential factor (thermodynamic factor) that decides the upper limit of specific capacity of HATBAQ, and the intermolecular stacking mode more affects the dynamic behavior (dynamic factor), such as rate performance.

To further improve the electrochemical properties of s-HATBAQ, it was compounded with GO via *in-situ* growth to obtain s-HATBAQ-rGOs. The chemical composition and electrochemical properties of the composites were explored in Figs S16–S22. Notably, s-HATBAQ-50% rGO presents four pairs of redox peaks and the highest integral charge area, suggesting more sufficient utilization of redox-active sites in s-HATBAQ-50% rGO than s-HATBAQ. This reason could be that the intrinsically conductive rGOs in the composites promote the charge transport throughout the bulk electrode as shown in Table S1 [44,45]. The electrochemical properties of s-HATBAQ-50% rGO at low current densities (0.1, 0.2, 0.3, and 0.5 A g^−1^) were also tested, and the corresponding reversible specific capacities are 405, 384, 365, and 359 mAh g^−1^, respectively (Fig. 2c). To determine whether rGO makes a contribution to the specific capacity, the electrochemical properties of rGO are presented in Fig. S23. rGO shows a typical capacitor's CV curve (Fig. S23a) and delivers a negligible specific capacity of 34 mAh g^−1^ at 1 A g^−1^ (Fig. S23b). The capacity contributions of rGO in the composites are 4, 15, and 34 mAh g^−1^ for s-HATBAQ-10% rGO, s-HATBAQ-30% rGO, and s-HATBAQ-50% rGO, respectively (Table S2). After subtracting the capacity contribution of rGO, the specific capacities of the active materials are 371, 350, 331, and 325 mAh g^−1^ at low current densities for s-HATBAQ-50% rGO, corresponding to 89, 84, 79.7, and 78.3% of the theoretical capacity, respectively, which (371, 350, 331, and 325 mAh g^−1^) are the highest reported values in organic electrodes of proton batteries so far. In addition, the *in-situ* growth method is more advantageous than simple physical mixture, which can be ascribed to the sufficient contact between s-HATBAQ and rGO (Fig. S24).

The long cycling performances of a-HATBAQ, s-HATBAQ, and s-HATBAQ-rGOs at current density of 20 A g^−1^ are shown in Fig. S25. After 5000 cycles, capacity retention of 100, 81.9, 77.5, 85.9, and 91.7% is achieved for a-HATBAQ, s-HATBAQ, s-HATBAQ-10% rGO, s-HATBAQ-30% rGO, and s-HATBAQ-50% rGO, respectively. Despite the high-capacity retention, the lower specific capacity of a-HATBAQ impedes its practical application. Furthermore, the FT-IR spectra (Fig. S26) and SEM images (Fig. S27) of s-HATBAQ-50% rGO remain almost unchanged after 5000 cycles at 20 A g^−1^, indicating good structural stability. Moreover, the s-HATBAQ-rGOs electrodes at different charge-discharge states were measured by *ex-situ* FT-IR spectra and XPS analysis (Fig. 2d–f). As depicted in Fig. 2d, the peak intensity of C−N (1131 cm^−1^) and C−O (1181 cm^−1^) stretching mode increases during discharge (Fig. 2e), and the vibration peak of the C=N bond at 1657 cm^−1^ and C=O bond at 1580 cm^−1^ gradually reduces with the increase in depth of discharge, suggesting that the C=N and C=O groups of s-HATBAQ convert to C−N and C−O [38]. High-resolution N1s and O1s XPS spectra further confirm the storage mechanism of s-HATBAQ electrodes in Fig. 2f. The intensity of C=N and C=O groups reduces, and the intensity of C−N and C−O bonds increases after discharging to −0.06 V and recovers to the original state after recharge. Those results demonstrate the reversible and stable electrochemical reactions based on the C=N and C=O bonds [40,46].

### Charge storage mechanism of s-HATBAQ and a-HATBAQ

**Figure 3.**
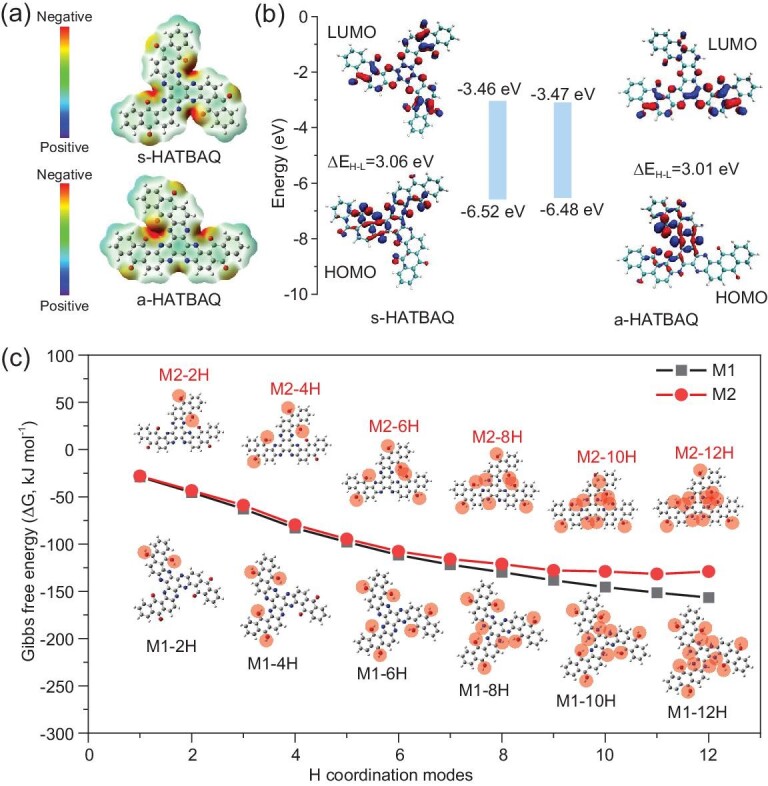
(a) Chemical structures and electrostatic potentials (ESP) of s-HATBAQ and a-HATBAQ. (b) HOMO/LUMO energy levels and energy gaps (ΔE_H-L_) of s-HATBAQ and a-HATBAQ. (c) The Gibbs free energy changes (ΔG) upon the complexation with H^+^ at different binding sites of s-HATBAQ and a-HATBAQ. The blue, red, black and gray balls represent the N, O, C and H atoms, respectively, and M1 and M2 stand for s-HATBAQ and a-HATBAQ, respectively.

Theoretically, s-HATBAQ and a-HATBAQ could accept 12 electrons based on the three pyrazine rings and six carbonyl groups (delivering a theoretical capacity of 415 mAh g^−1^). However, s-HATBAQ shows higher actual specific capacity than a-HATBAQ at the same current density in Fig. S11c and Fig. S10. Density functional theory (DFT) calculations were carried out to reveal the reason why the s-HATBAQ electrodes show a much better electrochemical performance (e.g. higher specific capacity and rate capacity) than a-HATBAQ. As depicted in Fig. 3a, the molecular electrostatic potential (ESP) shows that s-HATBAQ and a-HATBAQ own large electronegative regions and rich active sites for proton storage, which are mainly located around the C=O and C=N groups (Fig. 3a). Figure 3b shows the highest occupied molecular orbital (HOMO) and the lowest unoccupied molecular orbital (LUMO) of s-HATBAQ and a-HATBAQ. The extended conjugated structures of s-HATBAQ and a-HATBAQ endow them with relatively small band gaps (3.06 eV for s-HATBAQ and 3.01 eV for a-HATBAQ), suggesting that both of them are typical organic semiconductors, which was consistent with the experimental results shown in Table S3.

Furthermore, the protonation paths of s-HATBAQ and a-HATBAQ during the discharge process were calculated according to the minimum energy principle. The protonation processes of s-HATBAQ (M1) and a-HATBAQ (M2) are assumed to have six steps, and in each step two electrons and two H^+^ are combined. The charge storage mechanism of s-HATBAQ and a-HATBAQ is depicted in Figs S28 and S29. The optimal structures and Gibbs free energies of the protonated s-HATBAQ and a-HATBAQ molecules in each step are shown in Fig. 3c, Tables S4, and S5. Due to stronger electronegativity, the carbonyl group reacts preferentially with H^+^. Thus, the protonation process of s-HATBAQ and a-HATBAQ can be divided into two stages of carbonyl protonation and pyrazine protonation. Protons mainly react with three pairs of carbonyl groups in the first three steps, and then bond with pyrazines in the last three steps. Notably, for s-HATBAQ, the Gibbs free energy change (ΔG) gradually decreases with the increasing binding number of H^+^ and always remains negative, suggesting that s-HATBAQ could theoretically accept 12 H^+^ (12 electrons). It was supported by the fact that s-HATBAQ in s-HATBAQ-50% rGO electrodes present 89% utilization of redox-active sites (Fig. 2c). In contrast, although a-HATBAQ shows a slightly smaller band gap than s-HATBAQ, the ΔG of hydrogenation reaction from a-HATBAQ-11H to a-HATBAQ-12H turns positive due to strong steric hindrance, meaning the reaction will become nonspontaneous and easily incur a hydrogen evolution reaction before hydrogenation, which is consistent with the experimental results. Through the analysis on the hydrogenation processes of the two HATBAQ isomers, the stronger hindrance effect of a-HATBAQ against the proton insertion derives from its higher local active site density that can impede the entrance of protons more effectively by both physical blocking and electrostatic repulsion after partial hydrogenation, resulting in higher Gibbs free energy of hydrogenated products, and thus slow down or even stop the hydrogenation reaction. Additionally, the absolute value of ΔG for the hydrogenation of s-HATBAQ is larger than that of a-HATBAQ under the same protonation state, implying the stronger binding ability and probably faster reaction dynamics of s-HATBAQ with H^+^ than a-HATBAQ.

### Electrochemical performance of the full APBs at room temperature

The CF-KOH was prepared by soaking pristine carbon fibers (CFs) into 6 M KOH solution for 12 h, and exhibited significantly increased oxygen content and enhanced hydrophilicity (Fig. S30, Video S1 and Table S6), beneficial for the following MnO_2_ deposition. The MnO_2_@CF-KOH cathode was obtained according to our previous study [47]. The electrochemical properties, chemical information, and crystal structure of MnO_2_@CF-KOH as the cathode were evaluated using CV, XPS, and XRD in Figs S31 and S32. To elucidate which ion (H_3_O^+^ or Mn^2+^) plays the role of charge carrier, the CV curves and charge-discharge profiles of s-HATBAQ-50% rGO were investigated in 5 M H_2_SO_4_ and 5 M H_2_SO_4_ + 0.5 M Mn(BF_4_)_2_, respectively (Fig. S33). The CV and GCD curves of s-HATBAQ-50% rGO in the two different electrolytes show similar profiles, suggesting that H_3_O^+^ is the main charge carrier.

**Figure 4.**
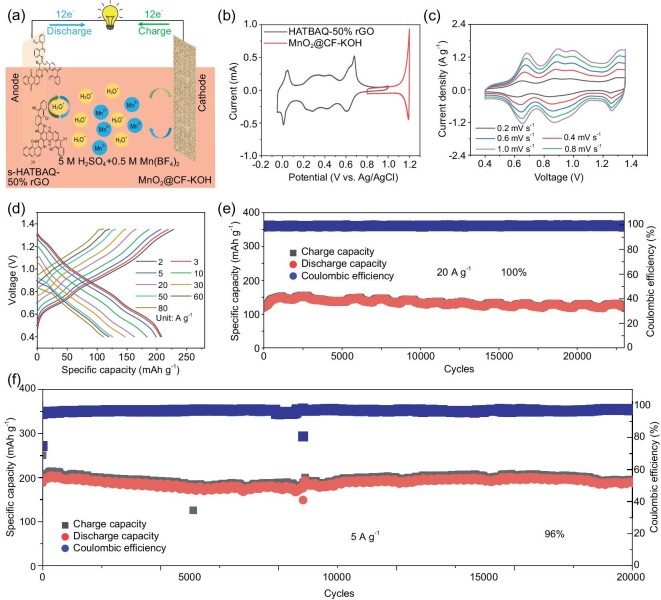
(a) Schematic illustration of the working mechanism for the MnO_2_@CF-KOH//s-HATBAQ-50% rGO. (b) CV curves of the s-HATBAQ-50% rGO anode (black line) and MnO_2_@CF-KOH cathode (red line) in 5 M H_2_SO_4_ + 0.5 M Mn(BF_4_)_2_ electrolyte. (c) CV curves of the MnO_2_@CF-KOH//s-HATBAQ-50% rGO at different scanning rates. (d) GCD curves of the MnO_2_@CF-KOH//s-HATBAQ-50% rGO at different current densities. (e and f) Cycle stability of the full battery tested at current densities of 20 A g^−1^ and 5 A g^−1^.

The electrochemical behavior of the s-HATBAQ electrode is characterized by the typical Swagelok cell, which is composed of a MnO_2_@CF-KOH cathode, a s-HATBAQ-50% rGO anode, and hybrid electrolyte (5 M H_2_SO_4_ + 0.5 M Mn(BF_4_)_2_) adsorbed with a glass fiber separator. Its operation depends on the MnO_2_/Mn^2+^ conversion in the cathode and H_3_O^+^ insertion/extraction in the anode (Fig. 4a, Equation 1, and Equation 2). The detailed reaction mechanism of MnO_2_ was discussed in Note S1.


(1) \begin{eqnarray*}
{\mathrm{6 M}}{{{\mathrm{n}}}^{{\mathrm{2 + }}}}{\mathrm{\ + \ 12 }}{{{\mathrm{H}}}_{\mathrm{2}}}{\mathrm{O}} \leftrightarrow {\mathrm{6 Mn}}{{{\mathrm{O}}}_{\mathrm{2}}}{\mathrm{\ + \ 24 H^+ \ + \ 12}}{{{\mathrm{e}}}^{\mathrm{ - }}}
\end{eqnarray*}

(2) \begin{eqnarray*}
&&{\mathrm{s}} \rm {-} \mathrm{HATBAQ\ + \ 12}{{{\mathrm{H}}}^{{\mathrm{ + \ }}}}{\mathrm{ + \ 12}}{{{\mathrm{e}}}^ - }\\
&&\quad \leftrightarrow \mathrm{s} \rm {-} \mathrm{HATBAQ} \rm {-} \mathrm{12H}
\end{eqnarray*}

Figure 4b presents CV curves of the s-HATBAQ-50% rGO anode (black line) and MnO_2_@CF-KOH cathode (red line) at 0.2 mV s^−1^. MnO_2_@CF-KOH//s-HATBAQ-50% rGO presents lower charge transfer resistance than MnO_2_@CF//s-HATBAQ-50% rGO in Fig. S34a, indicating that treating CFs with KOH could effectively promote Mn^2+^/MnO_2_ conversion. Moreover, Fig. S34b presents the cyclic voltammetry (CV) curves of MnO_2_@CF-KOH//s-HATBAQ-50% rGO and MnO_2_@CF//s-HATBAQ-50% rGO at 1 mV s^−1^ in a voltage range from 0.4 to 1.35 V. For the MnO_2_@CF-KOH//s-HATBAQ-50% rGO, three obvious redox peaks appear in the cathodic scans (0.62, 0.84, and 1.24 V) and the anodic scans (0.70, 0.92, and 1.32 V), suggesting that the intercalation and deintercalation of H_3_O^+^ into/out of s-HATBAQ-50% rGO are achieved in multiple steps. Furthermore, MnO_2_@CF-KOH//s-HATBAQ-50% rGO displays a larger peak current than MnO_2_@CF//s-HATBAQ-50% rGO, which can be ascribed to the lower charge transfer resistance of MnO_2_@CF-KOH//s-HATBAQ-50% rGO. To follow the nature of charge storage, the electrochemical reaction kinetics of MnO_2_@CF-KOH//s-HATBAQ-50% rGO battery were investigated by scanning CV at various sweep rates (Fig. 4c and Fig. S35a). For the MnO_2_@CF-KOH//s-HATBAQ-50% rGO battery, the b values of the three pairs of oxidation/reduction peaks were calculated to be 0.88/0.97, 0.81/0.97, and 0.73/0.86 (Fig. S35b), respectively, demonstrating a capacitive controlled process [48,49]. Figure S35c and S35d display the percentages of the capacitive contribution at various currents. As the scan rate increases from 0.2 to 1.0 mV s^−1^, the capacitive contribution ratios increase from 74.0% to 93.4%. The GCD curves and rate performance of the full battery at room temperature are displayed in Fig. 4d and Fig. S36, respectively. Highly reversible average discharge capacities of 210, 206, 200, 184, 163, 147, 126, 120, and 111 mAh g^−1^ were obtained at 2.0, 3.0, 5.0, 10.0, 20.0, 30.0, 50.0, 60.0, and 80.0 A g^−1^, respectively. The high specific capacity confers the MnO_2_@CF-KOH//s-HATBAQ-50% rGO cell with a high energy density of 178 Wh kg^−1^ at a power density of 1487 W kg^−1^ (based on the mass of s-HATBAQ). Furthermore, the MnO_2_@CF-KOH//s-HATBAQ-50% rGO battery shows an excellent cycling performance with a high capacity retention of 100% over 10 000 cycles at 10 A g^−1^ (Fig. S37) and 100% over 20 000 cycles at 20 A g^−1^ (Fig. 4e). At 5 A g^−1^, the battery delivers an initial specific capacity of 200 mAh g^−1^ and a capacity retention of 96% after 25 000 cycles (Fig. 4f), showing almost the best cycling stability among those recently reported proton batteries (Table S7). For example, MnO_2_@GF//TMBQ-rGO shows 77% capacity retention after 4000 cycles at 1.5 A g^−1^ [23]. MnO_2_@GF//PTO shows 80% capacity retention after 5000 cycles at 1 A g^−1^ [50]. The PCHL-rGO//Pb shows a capacity retention of 63% after 3000 cycles at 10 A g^−1^ [18]. The SEM images of electrodes after long cycling are shown in Fig. S38. The electrode morphology was not changed markedly and rGO wrapping structure could still be well observed even after 25 000 cycles at 5 A g^−1^. As a proof-of-concept demonstration, one Swagelok-type MnO_2_@CF-KOH//s-HATBAQ-50% rGO battery with 1.57 mg active material was able to illuminate a light emitting diode (LED) screen for over 20 seconds at 25°C (Video S2).

### Electrochemical performance of the full APBs at low temperature

It is well known that the physicochemical properties of the electrolyte are important for operating low-temperature proton batteries. Fluorine possesses stronger electronegativity than oxygen, and thus can form stronger hydrogen bonds with water molecules than that between water molecules, which will suppress the freezing of aqueous electrolyte at subzero temperatures, ensuring that the Mn(BF_4_)_2_-H_2_SO_4_ electrolyte can tolerate an ultralow ambient temperature. To further verify this mechanism, DFT and molecular dynamics (MD) simulations were conducted. As shown in Fig. S39a, Mn(BF_4_)_2_-H_2_O (−564.95 kcal mol^−1^) electrolyte exhibits lower E_binding energy_ than MnSO_4_-H_2_O electrolyte (−407.90 kcal mol^−1^), indicating that Mn(BF_4_)_2_ is more capable of breaking the H-bonds between water molecules. As shown in Fig. S39b, the MD simulation snapshots show that the introduction of Mn(BF_4_)_2_ causes breakage of H-bonds between water molecules and induces the formation of H-bonds between BF_4_^−^ and water molecules. Therefore, the saturated Mn(BF_4_)_2_-H_2_SO_4_ system shows a lower average number of H-bonds between water molecules than the saturated MnSO_4_-H_2_SO_4_ system (Fig. S39c). Above all, Mn(BF_4_)_2_-H_2_SO_4_ is selected to serve as the electrolyte of AHBs for achieving excellent freezing tolerance.

As shown in Fig. S40, the hybrid acid electrolyte (5 M H_2_SO_4_ + 0.5 M Mn(BF_4_)_2_) could still remain liquid even at −80°C for 48 h. Figure S41a exhibits the EIS spectra of the MnO_2_@CF-KOH//s-HATBAQ-50% rGO battery at different temperatures, and it can be seen that the first crossing point of Nyquist plot on Z′ axis shifts to the right with decreasing temperature, indicative of a negative correlation between the battery's internal resistance and the temperature. The ionic conductivities of the 5 M H_2_SO_4_ + 0.5 M Mn(BF_4_)_2_ electrolyte were also tested (Fig. S41b), exhibiting high values over the range of −80°C∼+25°C. Even at −80°C, the electrolyte still exhibited an impressive ionic conductivity (213.8 mS cm^−1^), which should be the highest reported value for acid electrolytes at such a low temperature so far. To reveal the evolution of ionic conductivity with temperature, the activation energy of the ionic conduction in the electrolyte was calculated based on the Arrhenius equation [51], and an ultra-small value of 0.078 eV was revealed (Fig. S42). Because of the exponential relationship between activation energy and ionic conductivity, the smaller activation energy implies the lower temperature dependence of ionic conductivity of the electrolyte, which is conducive for boosting its low-temperature performance. The high ionic conductivities and low activation energy of 5 M H_2_SO_4_ + 0.5 M Mn(BF_4_)_2_ facilitate the operation of proton batteries at low temperatures.

**Figure 5.**
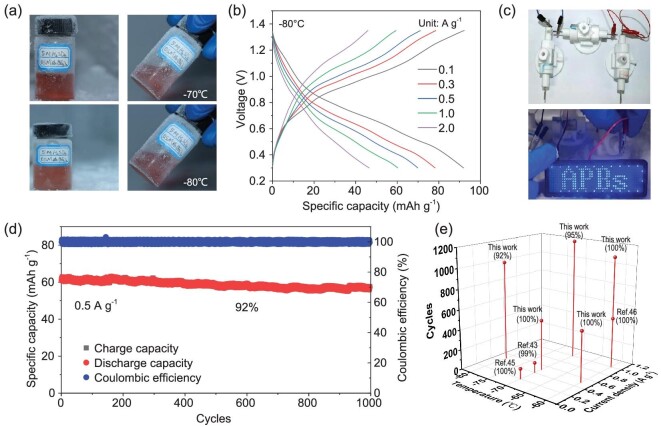
(a) The visual images of electrolyte at –70°C and −80°C. (b) GCD curves of MnO_2_@CF-KOH//s-HATBAQ-50% rGO battery at different current densities. (c) Optical image of an LED screen powered by the Swagelok-type MnO_2_@CF-KOH//s-HATBAQ-50% rGO battery at −80°C. (d) Long-term cycling stability of MnO_2_@CF-KOH//s-HATBAQ-50% rGO battery at −80°C. (e) Comparison of the cycling stability of the MnO_2_@CF-KOH//s-HATBAQ-50% rGO battery with the most previously reported low-temperature aqueous batteries.

The electrochemical properties of MnO_2_@CF-KOH//s-HATBAQ-50% rGO at low temperature are depicted in Fig. 5. As shown in Fig. 5a, 5 M H_2_SO_4_ + 0.5 M Mn(BF_4_)_2_ is still liquid at −70 and −80°C. This result demonstrates that the strong interaction between anions and water molecules breaks the hydrogen-bond network of water molecules and effectively suppresses the freezing of acidic electrolytes, thus guaranteeing the fast diffusion of H^+^ under subzero conditions. The electrochemical properties of the full APBs at low temperature are shown in Figs S43–S53 and Fig. 5b–e. Due to the excellent anti-freezing properties of the electrolyte, the full batteries retain excellent rate performance at −20 and −40°C (Fig. S45). Fig. S46 and Fig. 5b presents the specific capacities of the MnO_2_@CF-KOH//s-HATBAQ-50% rGO battery at −60, −70, and −80°C. Although the value reduces with decreasing temperature, a reversible capacity of 91.9. mAh g^−1^ (0.1 A g^−1^) was still achieved at −80°C. Moreover, three serial Swagelok full batteries (m_active material_ = 4.5 mg) can light up an LED for 10 seconds at −80°C, indicating that the MnO_2_@CF-KOH//s-HATBAQ-50% rGO batteries show promise for practical applications under extremely cold conditions (Fig. 5c, Video S3).

Additionally, a MnO_2_@CF-KOH//s-HATBAQ-50% rGO battery delivers excellent long cycling stability at −60 and −70°C. As depicted in Fig. S47, at −60°C, the battery presents a capacity retention of 100% after 1100 cycles at 1 A g^−1^ and 100% after 500 cycles at 0.5 A g^−1^. Even when the cycling was extended to 8900 cycles at 2 A g^−1^ (Fig. S48a) and 30 000 cycles at 5 A g^−1^ (Fig. S48b), the capacity retentions are 76.5% and 53.2%, respectively. The FT-IR spectra (Fig. S49) and SEM images (Fig. S50) of s-HATBAQ-50% rGO electrodes after cycling almost remain unchanged, indicating good structural stability. At −70°C, a capacity retention of 95% over 1200 cycles at 1 A g^−1^ and 100% after 500 cycles at 0.5 A g^−1^ can be obtained (Fig. S52). Even at −80°C, the full battery still possesses a capacity retention of 92% after 1000 cycles while maintaining an average discharge capacity of ∼60 mAh g^−1^ at 0.5 A g^−1^ (Fig. 5d), which is the best long cycling performance among all reported proton batteries under the same conditions (Fig. 5e, Table S7) [24,50,52,53]. Comparison of energy density and power density with the literatures is depicted in Table S8.

## CONCLUSION

In summary, s-HATBAQ exhibits significantly more excellent electrochemical properties than a-HATBAQ, due to the reduced steric hindrance and amorphous structure. s-HATBAQ-rGO composites with high electron conductivity and enhanced anti-corrosion properties were prepared via *in-situ* growth of s-HATBAQ on rGO, and they showed a record high specific capacity of 405 mAh g^−1^ at 0.1 A g^−1^ in 5 M H_2_SO_4_ electrolyte. The assembled full MnO_2_@CF-KOH//s-HATBAQ-50% rGO proton battery exhibits an exceptional cycle stability with a capacity retention of 96% after 25 000 cycles at 5 A g^−1^. In addition, an optimal anti-freezing electrolyte (5 M H_2_SO_4_ + 0.5 M Mn(BF_4_)_2_) was developed and could remain liquid even at −80°C, promising a high ionic conductivity of 213.8 mS cm^−1^. As a result, when operated at −80°C, the full battery exhibited a discharge-specific capacity of 91.9 mAh g^−1^ at 0.1 A g^−1^ and an outstanding long cycling stability with a capacity retention of 92% after 1000 cycles at 0.5 A g^−1^. This work describes the controllable synthesis and the structure-electrochemical property relationship of HATBAQ isomers, which not only inspires the study of high-performance wide-temperature-range proton batteries but also sheds light on molecule-level precise structure regulation for improving the performance of organic electrodes and promoting the development of energy storage.

## Supplementary Material

nwae045_Supplemental_Files
