# Supplementary material for: Steric hindrance modulation of hexaazatribenzanthraquinone isomers for high-capacity and wide-temperature-range aqueous proton battery
Source: Natl Sci Rev. 2024 Feb 1;11(4):nwae045. doi: 10.1093/nsr/nwae045 (PMC10972671; doi:10.1093/nsr/nwae045)
Supplement: nwae045_Supplemental_Files [file nwae045_supplemental_files.zip › Supplementary data.pdf]

# Steric hindrance modulation of hexaazatribenzanthraquinone isomers for high capacity and wide-temperature-range aqueous proton battery

*Mingsheng Yang,[1] Yuxin Hao,[2] Bei Wang,[2] Yan Wang,[3] Liping Zheng,[4] Rui Li,[2] Huige Ma,[2] Xinyu Wang,[2] Xiaoming Jing,[2] Hongwei Li,[1] Mengxiao Li,[1] Zhihui Wang,[1] Yujie Dai,[2] Guangcun Shan,[6] Mingjun Hu,[1]* Jun Luo,[5]* Jun Yang [2,5]**

1. School of Materials Science and Engineering, Beihang University, Beijing, 100191, China;
2. Beijing Institute of Nanoenergy & Nanosystems, Chinese Academy of Sciences, Beijing, 101400, China;
3. Center on Nanoenergy Research, School of Physical Science & Technology, Guangxi University, Nanning, 530004, China;
4. School of Chemistry and Chemical Engineering, Center on Nanoenergy Research, Guangxi University, Nanning 530004, China;
5. ShenSi Lab, Shenzhen Institute for Advanced Study, University of Electronic Science and Technology of China, Shenzhen, 518000, China;
6. School of Instrumentation Science and Opto-electronics Engineering, Beihang University, 100191, Beijing, China.

Corresponding authors:

mingjunhu@buaa.edu.cn, jluo@uestc.edu.cn, yangjun@binn.cas.cn

## 1. Materials and Methods

### 1.1 Materials

1,2-diamino-10-anthracenedione and hexaketocyclohexane octahydrate were obtained from MERYER. Acetic Acid, N-methyl-2-pyrrolidone (NMP), and methanol were purchased from MACKLIN. Oxide graphene was purchased from Dazhan Nano (Guangdong) Co., Ltd. All chemicals were used as received without further purification.

### 1.2 Preparation of symmetric HATBAQ

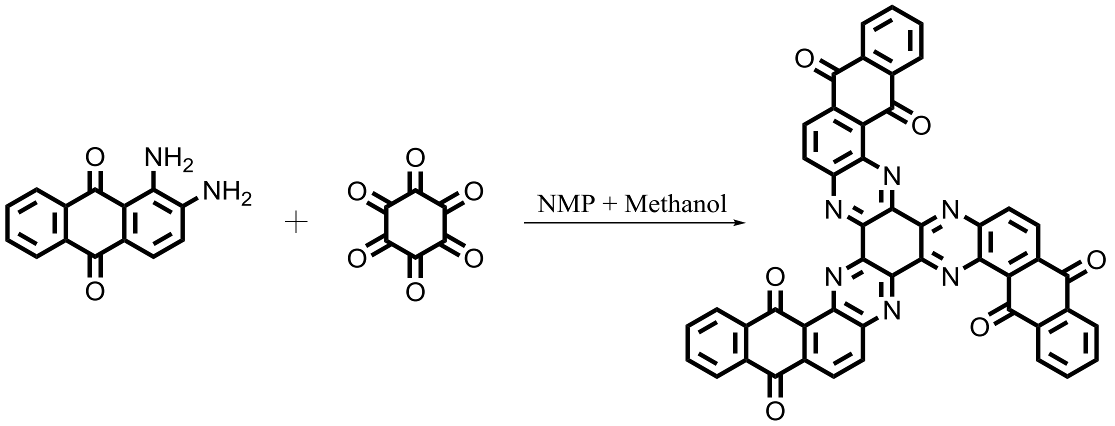


Scheme 1. Synthesis route for s-HATBAQ

Pressure resistant pipe with breather valve was charged with 1,2-diamino-10-anthracenedione (DMAD, 0.357g), hexaketocyclohexane octahydrate (HKH 8$H_2O$, 0.158 g), N-methyl-2-pyrrolidone (NMP, 20 mL) and methanol (5 mL). The sulfuric acid ($H_2SO_4$, 0.15 mL) was added dropwise to the mixture at 0 ℃ under stirring. Then, it was warmed up to 25 ℃, degassed by argon for 15 min, stirred for 2 h, and kept at 155 ℃ for 24 h. NM-155 (0.24 g) were obtained by careful filtration, washing with methanol and water, followed by drying at 120 $^{o}$C under reduced pressure for 1 day. NM-115, NM-135, and NM-175 were prepared according to similar method to s-HATBAQ-155, except adjusting reaction temperature to 115, 135, or 175 ℃. During the reaction process, concentrated sulfuric acid can provide a large amount of hydrogen ions, which protonate the carbonyl oxygen of hexaketocyclohexane, making the carbonyl oxygen and carbon atoms more positively charged, thus facilitating the nucleophilic attack of amine compounds. The yield of

s-HATBAQ was 70 %.

**1.3 Preparation of asymmetric HATBAQ**

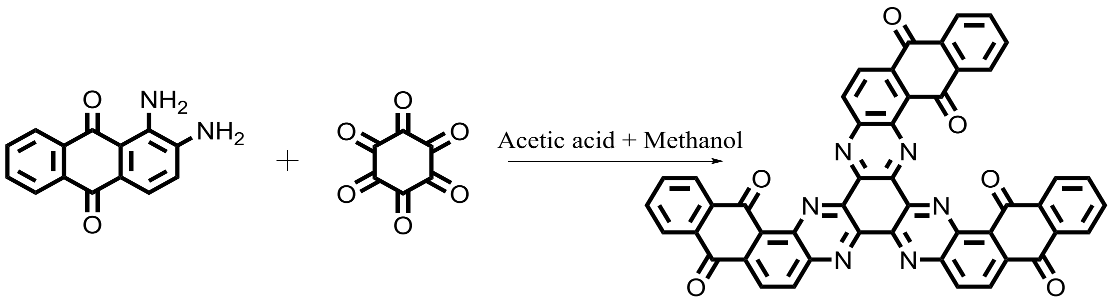


Scheme 2. Synthesis route for a-HATBAQ

Pressure resistant pipe with breather valve was charged with 1,2-diamino-10-anthracenedione (DMAD, 0.357g), hexaketocyclohexane octahydrate (HKH 8$H_2O$, 0.158 g), Acetic acid (20 mL) and methanol (5 mL). Then, it was degassed by argon for 15 min, stirred for 2 h, and kept at 120 ℃ for 24 h. AM-120 were obtained by careful filtration, washing with methanol and water, followed by drying at 120 $^{o}$C under reduced pressure for 1 day. The yield of a-HATBAQ was 75 %.

**1.4 Preparation of s-HATBAQ-rGO composites**

s-HATBAQ-rGO composites were prepared in the same condition to s-HATBAQ-155, but with the addition of oxide graphene (0.026g, 0.103g, 0.24g) and denoted as s-HATBAQ-10% rGO, s-HATBAQ-30% rGO, and s-HATBAQ-50% rGO, respectively. s-HATBAQ/50% rGOs were prepared by physically grinding s-HATBAQ (60 mg) and reduced oxide graphene (rGO, 60 mg) for 1h. rGO (38mg) was prepared by treating GO (40 mg) in the mixture of NMP and methanol at 155℃ for 24 h.

**1.5 Preparation of CF-KOH**

Commercial carbon fiber (CFs) with thickness of 5 cm were firstly cut into desired size (1 cm × 2 cm), immersed in acetone solution at room temperature for 24 h. Next, CFs were ultrasonic washed with deionized water and ethanol for several times to remove acetone. Then, they were soaked in 6 M potassium hydroxide (KOH) solution for 12 h, and putted into a low temperature refrigerator at -80 ℃ for 4 h, and

then freeze-dried for 2 days. Finally, CFs were heat-treated at 800 ℃ for 2 h with a ramping rate of 3℃/min at argon atmosphere. After naturally cooling down to room temperature, CFs were washed with plenty of water to neutral, and then dried at 80 ℃ for 24 h under reduced pressure.

**1.6 Preparation of electrode materials**

The s-HATBAQ (a-HATBAQ)-based electrode was prepared by mixing active material (HATBAQ) with conductive additive and binder (PVDF) in a mass ratio of 60:30:10 in N-Methylpyrrolidone (NMP) solvent, and coated the slurry onto carbon cloth. Then the electrode films were dried at 80 ℃ for 12 h under vacuum. HATBAQ-rGO electrodes and HATBAQ/50% rGO were prepared by mixing active material with conductive additive and binder (PVDF) in a mass ratio of 70:20:10 in N-Methylpyrrolidone (NMP) solvent, and coated the slurry onto carbon cloth. Then the electrode films were dried at 80 ℃ for 12 h under vacuum. The active material mass loadings of s-HATBAQ and a-HATBAQ electrodes were about 1.0-1.3 mg and 1.1-1.4 mg, respectively. The active material mass (s-HATBAQ) loadings of s-HATBAQ-rGO electrodes were about 1.0-1.2 mg. The active material mass (s-HATBAQ) loadings of s-HATBAQ/50% rGO were about 1.0-1.4 mg. Activated carbon (AC) membrane was prepared by mixing activated carbon with Ketjen black and PVDF in a mass ratio of 80:10:10 in NMP, coated the slurry onto carbon paper, and dried at 80 ℃ for 12 h under vacuum. A piece of CF-KOH (1.0 $cm^2$) with pre-electrodeposited 15 mAh $MnO_2$ ($MnO_2$@CF) under a constant current density of 5 mA $cm^{-2}$ for 3 h was used as the cathode. It should be noted that pre-electrodeposited $MnO_2$ in CF or CF-KOH can enhance the coulombic efficiency of the full battery.

**1.7 Electrochemical tests**

The electrochemical properties of s-HATBAQ, a-HATBAQ, and s-HATBAQ-rGOs electrodes were tested via three electrode device using s-HATBAQ, a-HATBAQ, and s-HATBAQ-rGOs electrodes as working electrodes, activated carbon as the counter electrode, silver/silver chloride electrode (Ag/AgCl) as the reference electrode, and 5 M $H_2SO_4$ as the electrolyte. The electrochemical properties

of $MnO_2$@CF-KOH were tested according to the same method as that of s-HATBAQ, but using 5 M $H_2SO_4$+0.5 M $Mn(BF_4)_2$ as the electrolyte. The electrolyte is aerated with nitrogen for half an hour to remove oxygen. The full battery was assembled using s-HATBAQ-50% rGOs as the anode and $MnO_2$@CF-KOH as the cathode, which was tested by swagelok cell systems. The cyclic voltammetry (CV) measurements were performed on an AMETEK-Princeton Applied Research electrochemical workstation. The galvanostatic charge-discharge (GCD) tests were conducted on a LAND-CT2001A battery testing instrument, and the capacities are calculated based on the mass of the active materials (s-HATBAQ) in the anode. The pictures of electrolytes at different temperatures were taken after being frozen at desired temperatures for 2 h. The batteries were kept at a specified temperature for 2 h before charge-discharge tests to ensure the battery temperature equivalent to the setting temperature of the freezer.

**2. Materials Characterizations.** Fourier Transform Infrared (FT-IR) spectrum was measured on a VERTEX80v (Bruker, German) with Universal Attenuated Total Reflection (ATR) accessory between the range of 400-4000 $cm^{-1}$. Material morphology was characterized by scanning electron microscopy (FEI Nova Nano SEM, American) and transmission electron microscopy (TEM, Tecnai G2 F30; 200 kV) analyses. X-ray photoelectron spectroscopy (XPS) was attained using Thermo Scientific Escalab 250Xi. The electronic conductivity of samples was tested by the quadrupole probe method (ST2722-SD). The $^{13}C$ nuclear magnetic resonance (NMR) were conducted on a 600 MHz NMR spectrometer (VNMRS, 600 MHz). The m/z of HATBAQ was tested by Matrix-Assisted Laser Desorption/ Ionization Time of Flight Mass Spectrometry (Thermofisher, American). Contact angle testament was tested by Dataphysics OCA20 (German). Powder X-ray Diffraction (PXRD): PXRD patterns were collected on Xpert3 Powder. Thermogravimetric analysis (TGA) was performed using a NETZSCH STA 449 $F_5/F_3$ Jupiter thermogravimetric analyzer at a ramping rate of 10 ℃ $min^{-1}$ to 900 ℃ at the atmosphere of $N_2$.

**Calculation method**

**Material characterization**

These structures of these reactants and products were all optimized under the framework of density of functional theory (DFT) with m062x functional **[1]** and 6-31g(d) **[2, 3]** basis set. The vibrational frequency analysis was carried out for the optimized structure with the same calculation method. The thermodynamic correction terms of there structures at 298.15K were then obtained using Shermo program **[4]**. In order to obtain the electron energy with higher accuracy which has the major impact on the accuracy of Gibbs free energy, a single point calculations for these optimized structures with m062x functional and 6-311+G(d,p) basis set were performed. Finally, the single point energy was added to the free energy correction calculated before to obtain the Gibbs free energy. All these DFT calculations were performed using Gaussian 16 program suite **[5]**. The visualization of the frontier molecular orbitals were rendered using Visual Molecular Dynamic program (VMD) **[6]**.

**Electrolyte characterization**

Molecular dynamics (MD) simulations are performed with Gromacs version 2019.6 **[7]**. Initial simulation system of $Mn(BF_4)_2$ system is constructed by mixing 30 $Mn^{2+}$, 600 $H_3O^+$, 300 $SO_4^{2-}$, 60 $BF_4^-$ with 3300 water molecules. In the system of $MnSO_4$, $BF_4^-$ ions are excluded and 30 extra $SO_4^{2-}$ are added. TIP3P model **[8]** is used for water. For $H_3O^+$, we use parameters suggested by Hristov et al. **[9].** For $Mn^{2+}$, parameters proposed by Merz et al.**[10]** are adopted. For $SO_4^{2-}$, parameters proposed by Williams et al. are used **[11]**. For $BF_4^-$, the universal force field **[12]** in combination with general amber force field **[13]** are used. Van der Waals interactions are computed by Lennard Jones (LJ) potential truncated at 1.2 nm. Coulombic interactions are calculated with PME method. 50-ns production runs are conducted at 298.15 K and 1 bar. Three-dimensional periodic boundary conditions are always considered. Binding energy are determined by quantum chemical calculations with Gaussian 16 software, at the B3LYP-D3(BJ)/6-311G** level of theory.

**Theoretical specific capacity:**

$$C=\frac{n\times F}{M_W}=\frac{n\times 96485}{3.6\times M_W} \qquad (1)$$

Where n is the number of electrons transferred per redox reaction. F represents the Faraday constant, and Mw is the molar weight of the organic component. The molecular weight of HATBAQ is 774.71 g mol$^{-1}$ ($C_{48}H_{18}N_6O_6$). The number of transferred electrons (n) of HATBAQ are 12. Therefore, using Equation S1, the theoretical capacity of s-HATBAQ is calculated to be 415 mAh g$^{-1}$.

**Calculation Details:** The ionic conductivity is calculated following the equations

$$\sigma=\frac{L}{RS} \qquad (2)$$

where σ is the ionic conductivity, L is the thicknesses of the electrolyte, R is the resistance of the electrolyte, and S is the area of the electrodes.

The energy density and power density of the $MnO_2$@CF-KOH//s-HATBAQ-50% rGO cell were calculated by the following equations:

$$E=\frac{C\times V}{m} \qquad (3)$$

$$P=\frac{E}{t} \qquad (4)$$

where E represents the energy density (Wh kg$^{-1}$), C is the discharge capacity of the cell (mAh), V is the average discharge voltage of the cell (V), and m is the total mass (kg) of the anode (s-HATBAQ). P represents the power density (W kg$^{-1}$), and t is the time for full discharge (h).

$$\sigma=\frac{A}{T}e^{-\frac{E_a}{KT}} \qquad (5)$$

$$\text{In}(\sigma T)=\text{In}(A)-\frac{E_a}{KT} \qquad (6)$$

σ is ionic conductivity, T is temperature, Ea is the activation energy, and k is Boltzmann constant.

Galvanostatic intermittent titration technique (GITT) measurement was performed to determine the $H_3O^+$ diffusion coefficient in a-HATBAQ and s-HATBAQ electrodes during a discharge charge process in 5 M $H_2SO_4$, with a current pulse at 0.08 A g$^{-1}$ for 600 s followed by a relaxation period for 2400 s. The diffusion coefficient of $H_3O^+$ in a-HATBAQ and s-HATBAQ electrodes can be obtained according to the following equation.

$$D=\frac{4}{\pi\tau}\left(\frac{n_m V_m}{S}\right)^2\left(\frac{\Delta E_s}{\Delta E_t}\right)^2 \qquad (7)$$

Where $\tau$ is the relaxation time, $n_m$ and $V_m$ refer to the number of moles and the molar volume of active electrode material, respectively. ΔEt represents the total voltage change during the current pulse time $\tau$ without IR drop, and ΔEs stands for the voltage difference between the equilibrium states before and after the current pulse.

As shown in **Figure S1b**, the MALDI-TOF-MS confirms the presence of the substances with the same molecular weight as the target molecules (M=774.71) in the five products (For NM-135, NM-155, and NM-175, the m/z values of 746.8, 718.8, and 690.8 may be attributed to the loss of $[C_2H_4]^+$, 2 $[C_2H_4]^+$, and 3 $[C_2H_4]^+$ from HATBAQ).

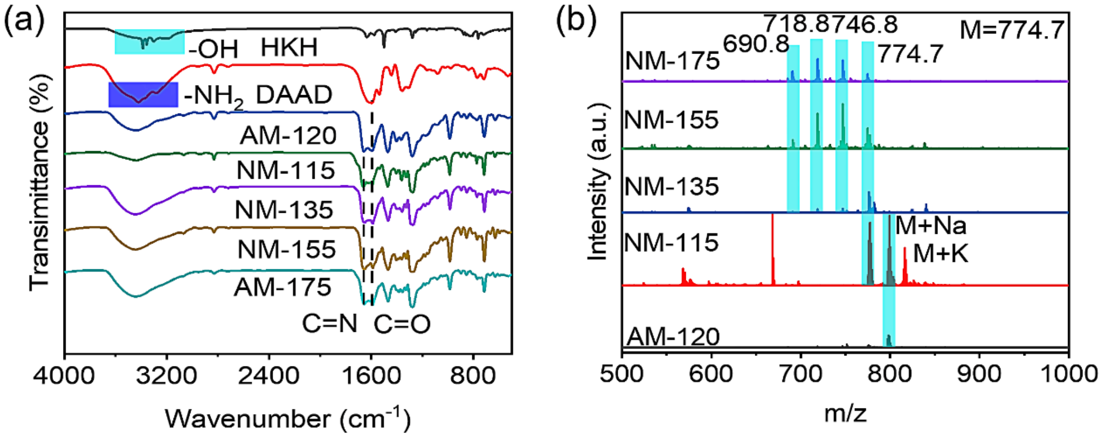


Figure S1. (a) FT-IR spectra of HKH, DAAD, AM-120, NM-115, NM-135, NM-155 and NM-175, (b) Mass spectra of NM-115, NM-135, NM-155, and NM-175.

As shown in **Figure S2**, Notably, for NM-115, NM-135, NM-155, and NM-175, as the reaction temperature increases from 115 to 155 ℃, the total number of carbon chemical shift peaks decreases, indicating the reduction of impurity in products (**Figure S2**). Moreover, when the reaction temperature was 175 ℃, NM-175 exhibits ten peaks, which may be because that the high temperature causes side reactions in the reaction. Thus, the optimal reactional temperature for synthesis of s-HATBAQ was 155 ℃.

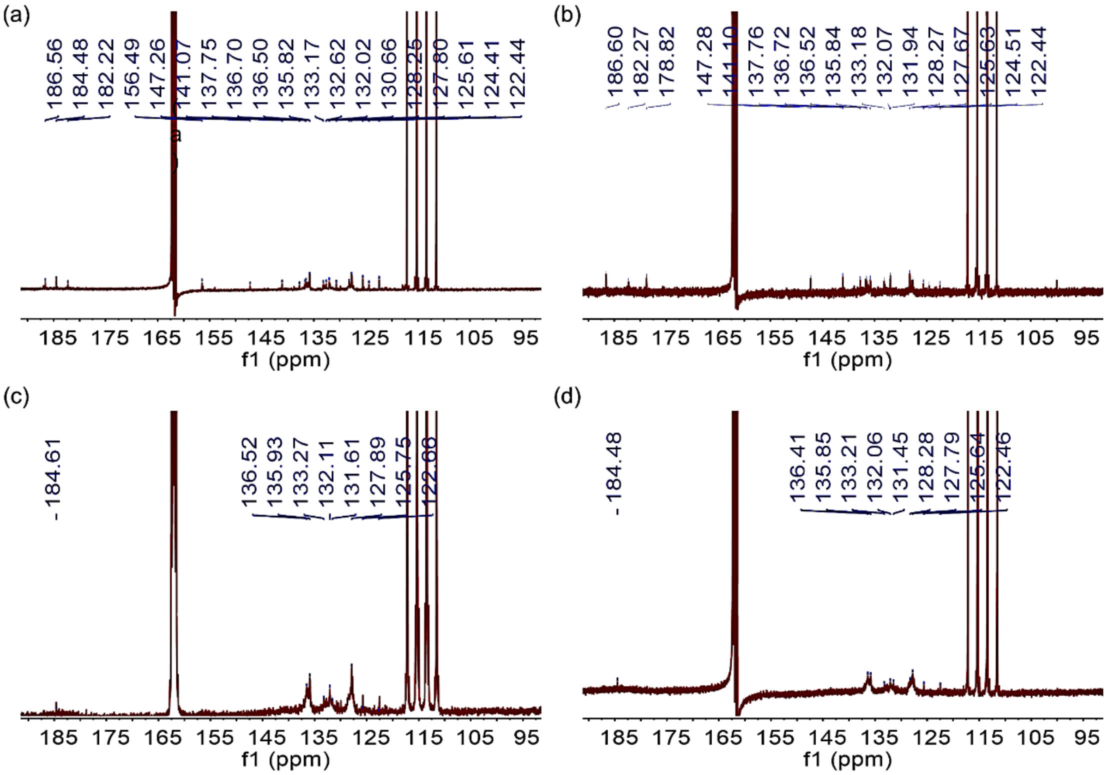


Figure S2. $^{13}$C NMR spectra of (a) NM-115, (b) NM-135, (c) NM-155, and (d) NM-175

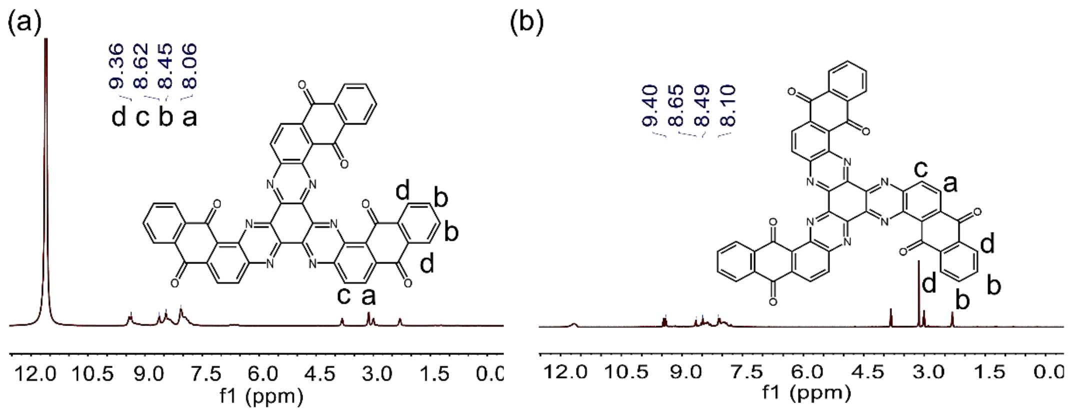


Figure S3. $^{1}$H NMR spectra of (a) AM-120 and (b) NM-155

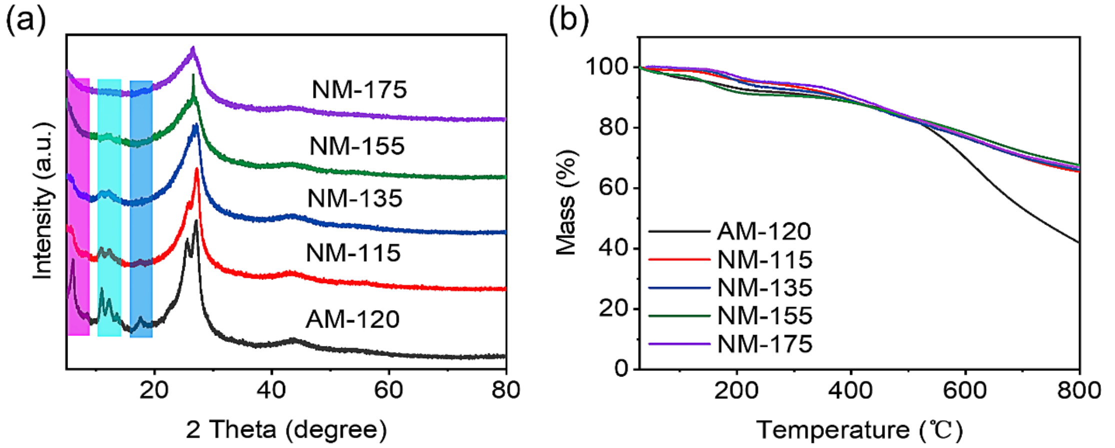


Figure S4. (a) XRD patterns of AM-120, NM-115, NM-135, NM-155 and NM-175, and (b) TGA curves of AM-120, NM-115, NM-135, NM-155 and NM-175

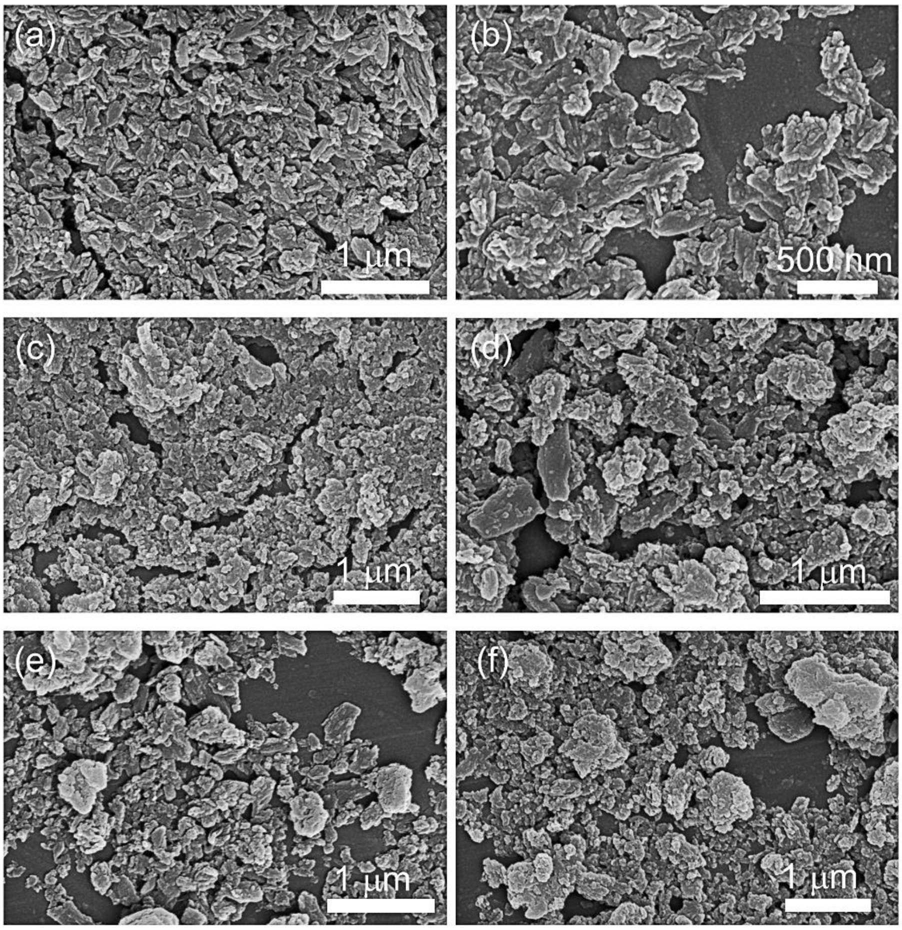


Figure S5. SEM images of (a,b) AM-120, (c) NM-115, (d) NM-135, (e) NM-155, and (f) NM-175.

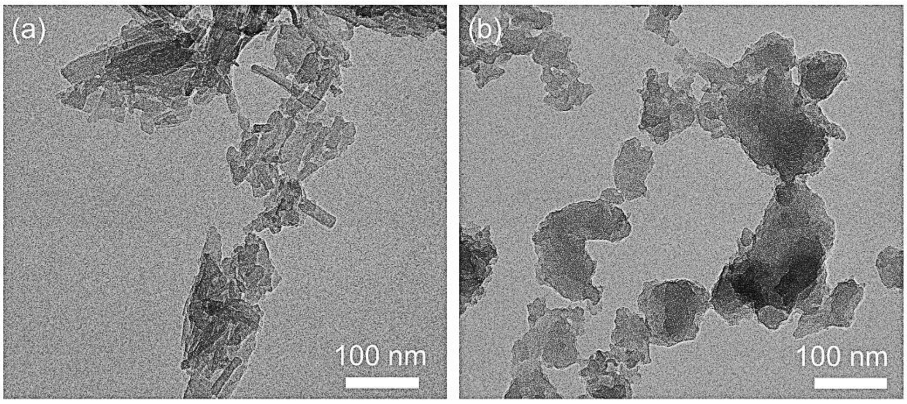


Figure S6. TEM images of (a) AM-120 and (b) NM-155.

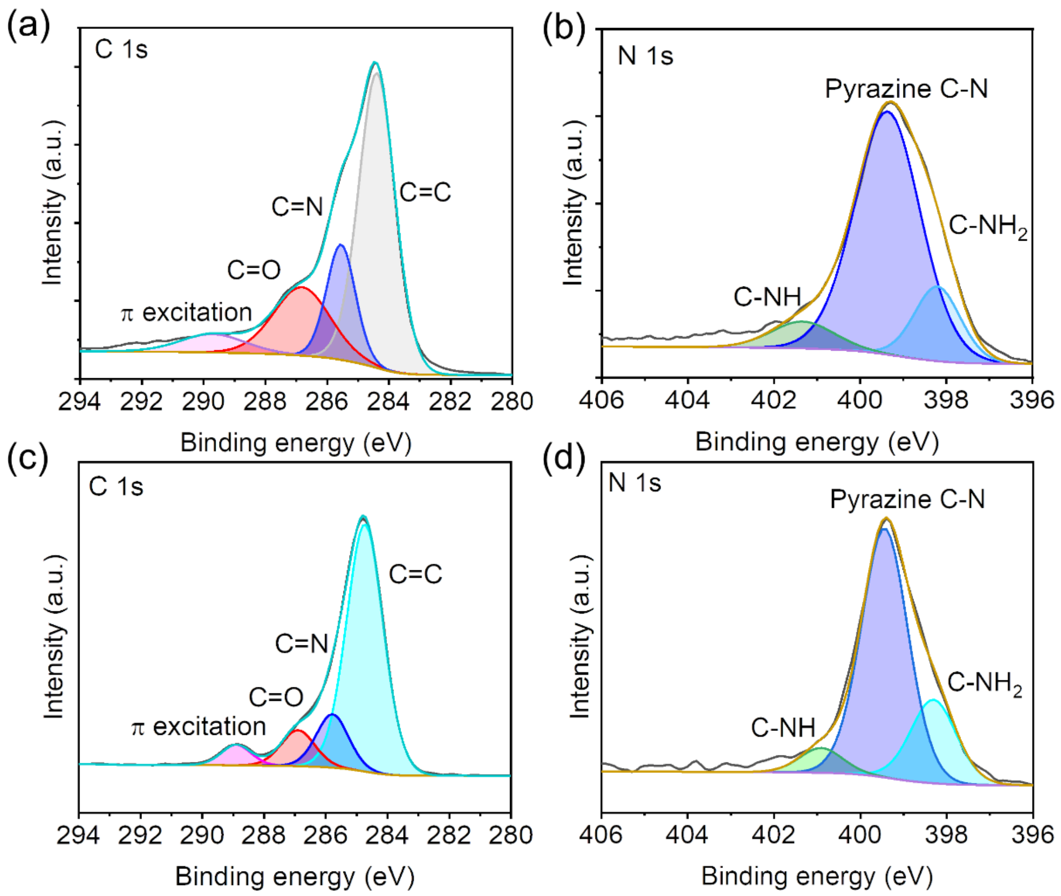


Figure S7. (a,b) C 1s and N 1s XPS spectra of NM-155 and (c,d) C 1s and N 1s XPS spectra of AM-120

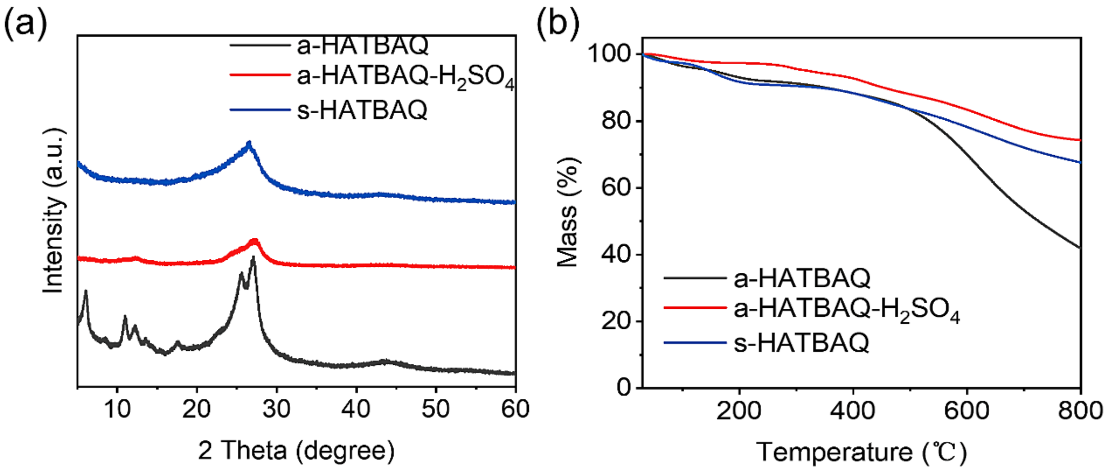


Figure S8. (a) XRD patterns and (b) TG curves of a-HATBAQ, a-HATBAQ-$H_2SO_4$, and s-HATBAQ.

The electrochemical properties of the five products were also investigated in 5 M $H_2SO_4$. Their cyclic voltammetry (CV) curves at 1 mV $s^{-1}$ were shown in **Figure S9**. As shown in **Figure S9c-9f** NM-115, NM-135, NM-155, and NM-175 show four pairs of redox peaks, while AM-120 can only display three pairs of redox peaks. To elucidate the interesting phenomenon, DFT was adopted. As shown in Figure 3c, for s-HATBAQ, the Gibbs free energy change ($\Delta$G) gradually decreases with increasing binding number of $H^+$ and always remains negative, suggesting that s-HATBAQ could theoretically accept 12 $H^+$ (12 electrons). In contrast, although a-HATBAQ shows a slightly smaller band gap than s-HATBAQ, the ΔG of hydrogenation reaction from a-HATBAQ-11H to a-HATBAQ-12H turns positive due to the strong steric hindrance. Due to strong steric hindrance, a-HATBAQ can only accept 11 $H^+$ (11 electrons) and display three redox peaks. For NM-x samples, with the reaction temperature increasing from 115 ℃ to 155 ℃, hydrogen evolution at low potential is gradually relieved, and from 155 ℃ to 175 ℃, hydrogen evolution becomes a little more serious again. Especially NM-155 exhibits an obvious reduction peak at about 0 V (vs. Ag/AgCl), implying its hydrogen evolution was inconspicuous at such potential (**Figure S9e**).

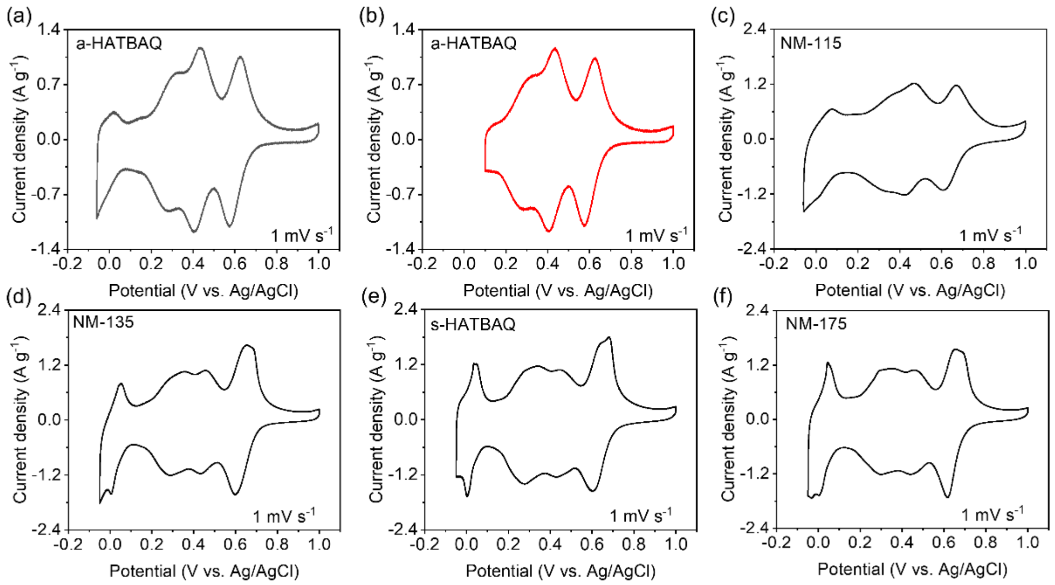


Figure S9. CV curves at 1 mV $s^{-1}$ of (a) and (b) a-HATBAQ, (c) NM-115, (d) NM-135, (e) s-HATBAQ, and (f) NM-175.

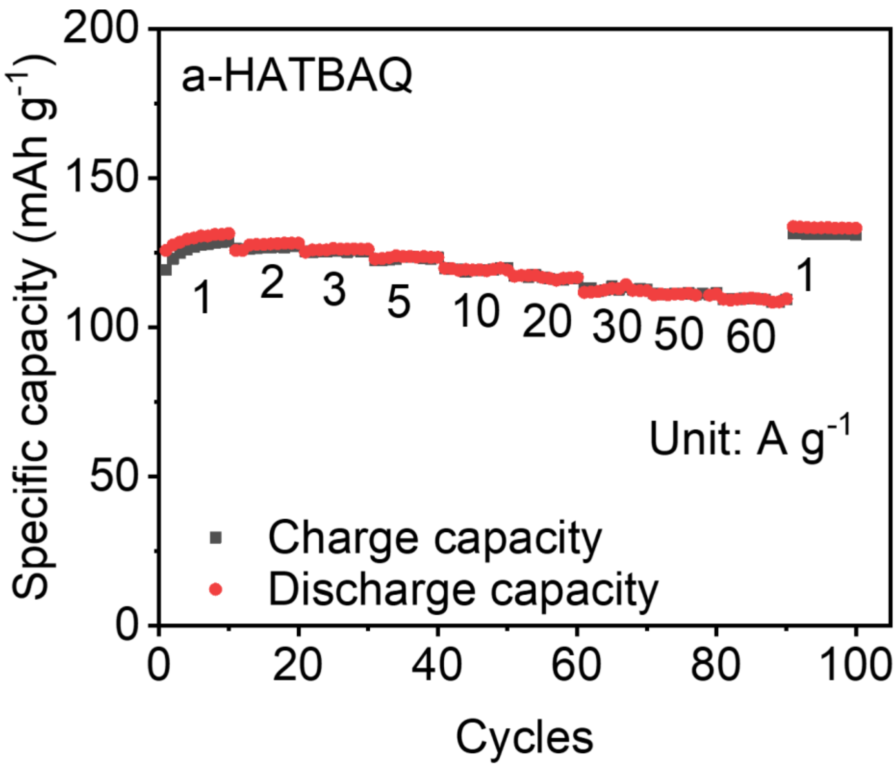


Figure S10. Rate performance of a-HATBAQ

The significant hydrogen evolution rule should be reflected from the molecular microstructure evolution. Rate performance of AM-120, NM-115, NM-135, NM-155, and NM-175 are depicted in **Figure S10** and **Figure S11a-d**. At 1 A $g^{-1}$, the coulombic efficiencies of NM-115, NM-135, and NM-175 exceed 110 %, much higher than s-HATBAQ-155 (106 %), due to the enhancive hydrogen evolution. NM-155 also shows better rate capability than AM-120 (**Figure S11c and Figure S10**), and the discharge capacities are 202, 170.5, and 155.2 mAh $g^{-1}$ at 1, 20, and 60 A $g^{-1}$, respectively, much higher than those of a-HATBAQ at same current density (131.5, 117.8, and 109.5 mAh $g^{-1}$). These results reveal that NM-115, NM-135, NM-155, and NM-175 have wider voltage windows and higher specific capacities than AM-120 in $H_2SO_4$ electrolyte. It is thought that unfavorable steric hindrance and serious hydrogen evolution side reaction of AM-120 cause lower utilization of active sites and thus lower specific capacity.

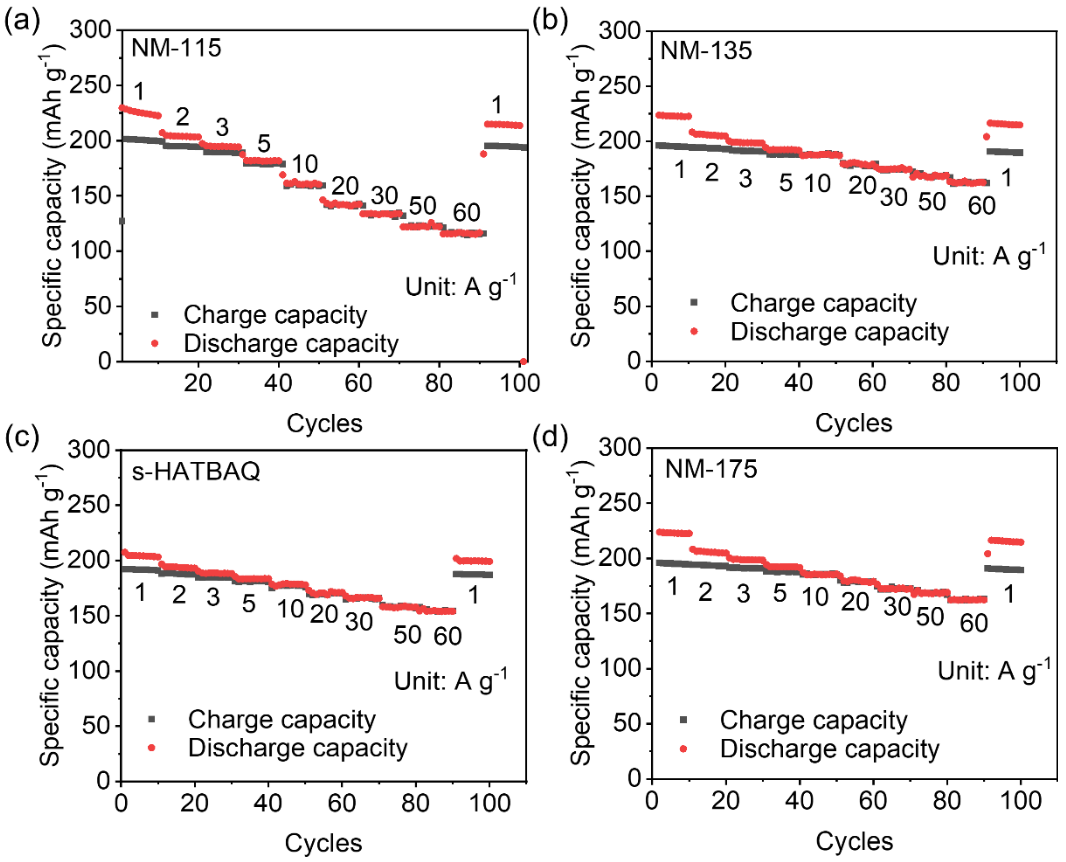


Figure S11. Rate performance of (a) NM-115, (b) NM-135, (c) s-HATBAQ, and (d) NM-175.

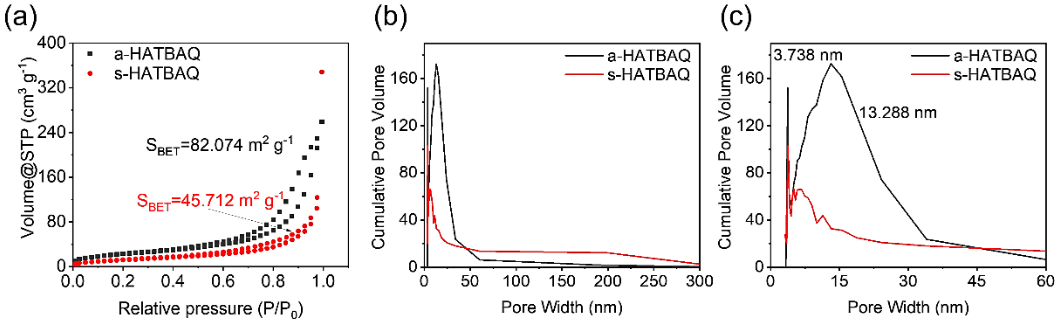


Figure S12. (a) Nitrogen adsorption and desorption isotherms, (b) pore size distribution, and (c) pore size distribution of a-HATBAQ and s-HATBAQ

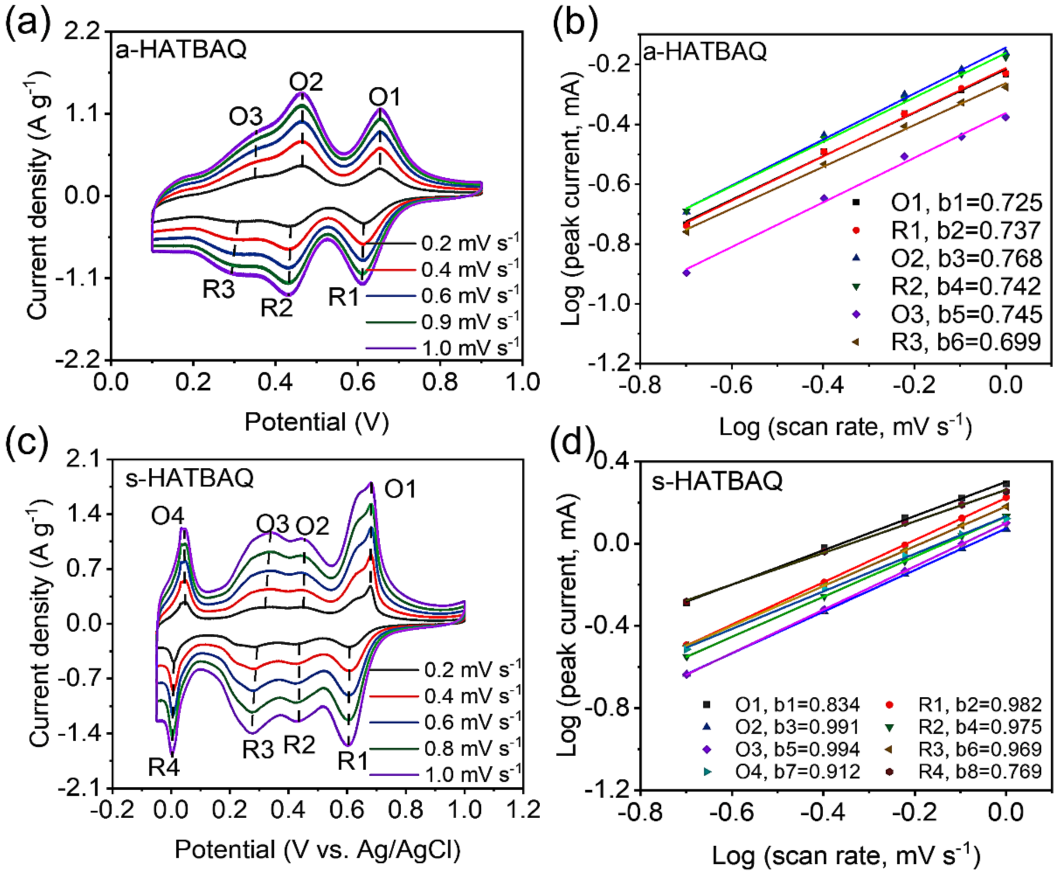


Figure S13. (a) CV curves at different scan rates of a-HATBAQ, (b) the corresponding plots of log (i) versus log (v) at each redox peak of a-HATBAQ, (c) CV curves at different scan rates of s-HATBAQ, and (d) the corresponding plots of log (i) versus log (v) at each redox peak of s-HATBAQ.

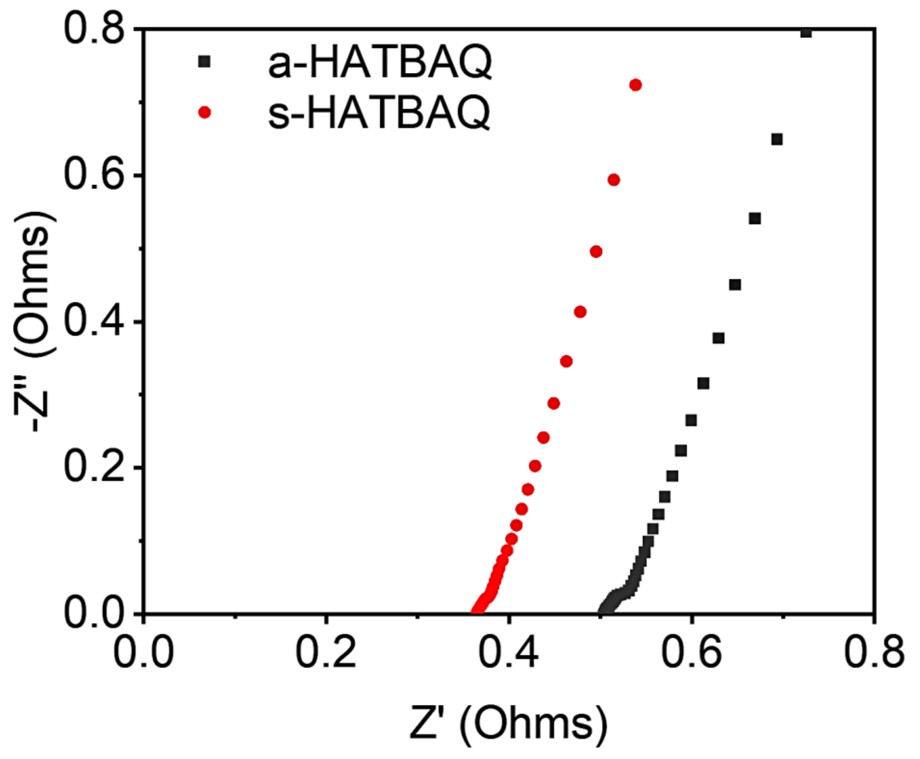


Figure S14. EIS values of a-HATBAQ and s-HATBAQ

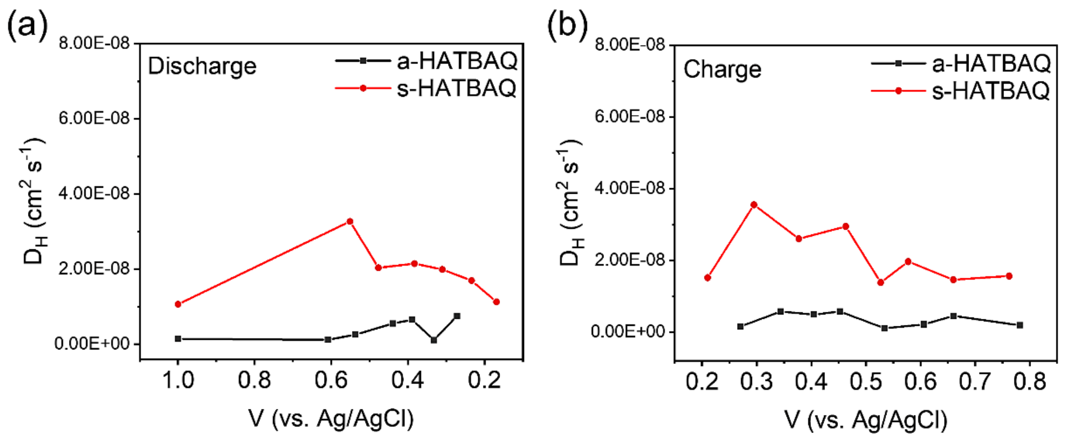


Figure S15. GITT curves and the calculated apparent diffusion coefficient of $H^+$ of a-HATBAQ and s-HATBAQ under (a) discharge, and (b) charge process

As shown in **Figure S16**, the characteristic XRD peak of GO at 10.6 ° is not found in s-HATBAQ-10% rGO, s-HATBAQ-30% rGO, and s-HATBAQ-50% rGO, indicative of the reduction of GO.

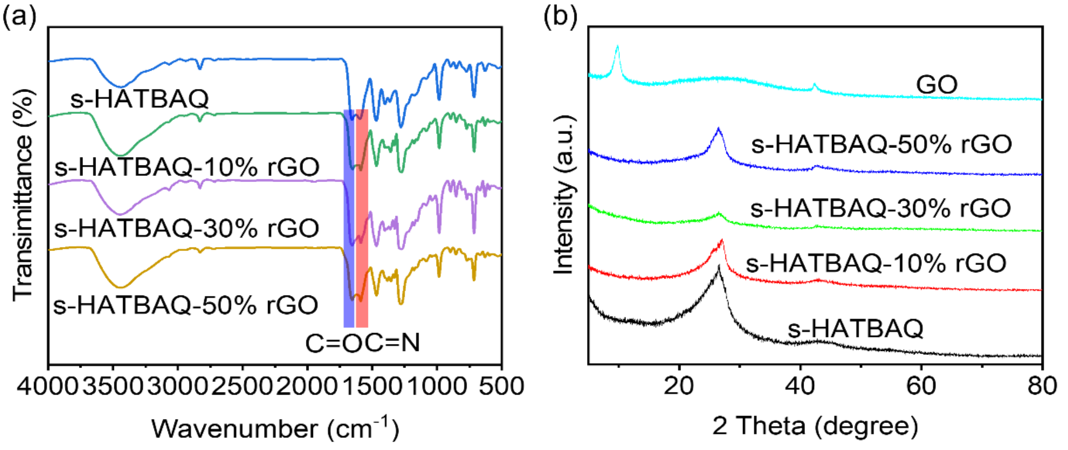


Figure S16. (a) FT-IR spectra and (b) XRD patterns of s-HATBAQ and s-HATBAQ-rGOs.

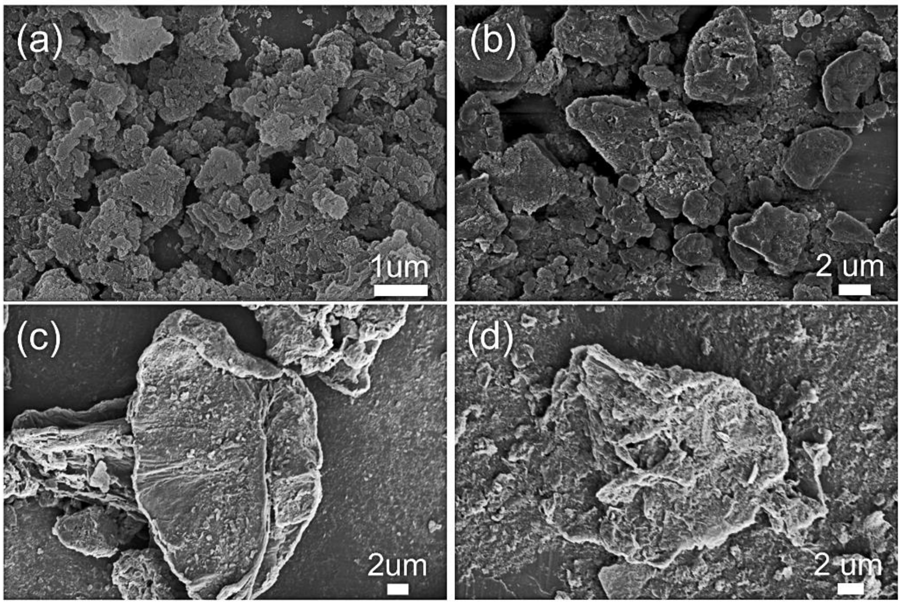


Figure S17. SEM images of (a) s-HATBAQ, (b) s-HATBAQ-10% rGO, (c) s-HATBAQ-30% rGO, and (d) s-HATBAQ-50% rGO.

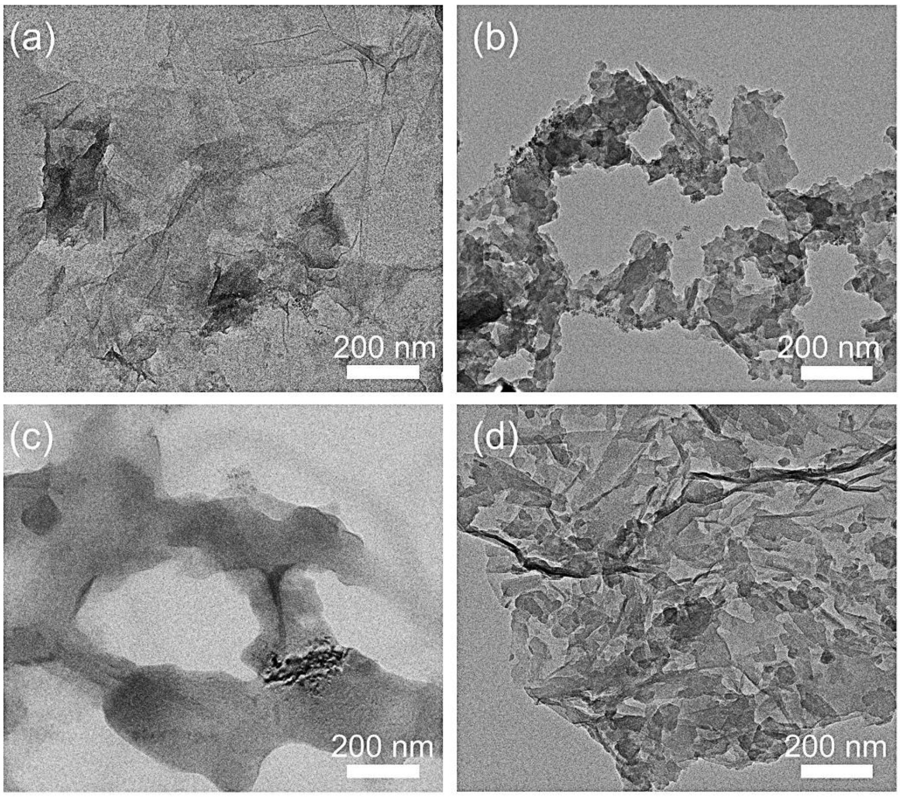


Figure S18. HR-TEM image and selected-area electron diffraction pattern of (a) GO, (b) s-HATBAQ-10% rGO, (c) s-HATBAQ-30% rGO, and (d) s-HATBAQ-50% rGO.

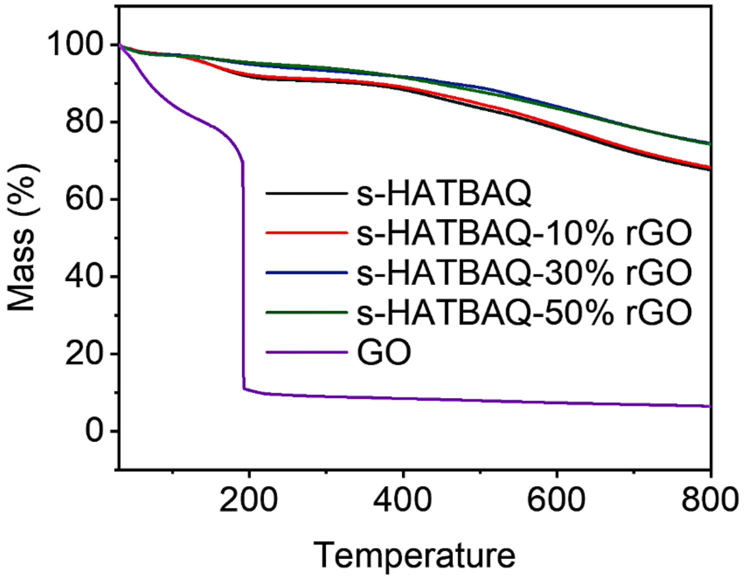


Figure S19. TG curves of s-HATBAQ, s-HATBAQ-rGOs, and GO.

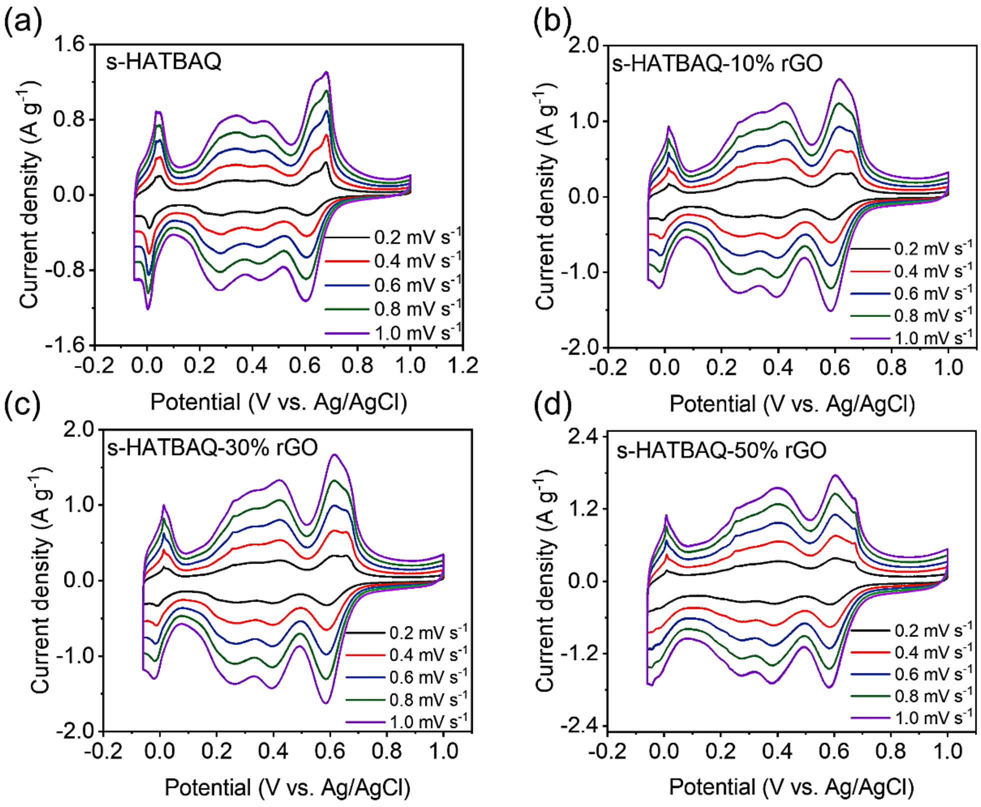


Figure S20. CV curves of (a) s-HATBAQ, (b) s-HATBAQ-10% rGO, (c) s-HATBAQ-30% rGO, and (d) s-HATBAQ-50% rGO.

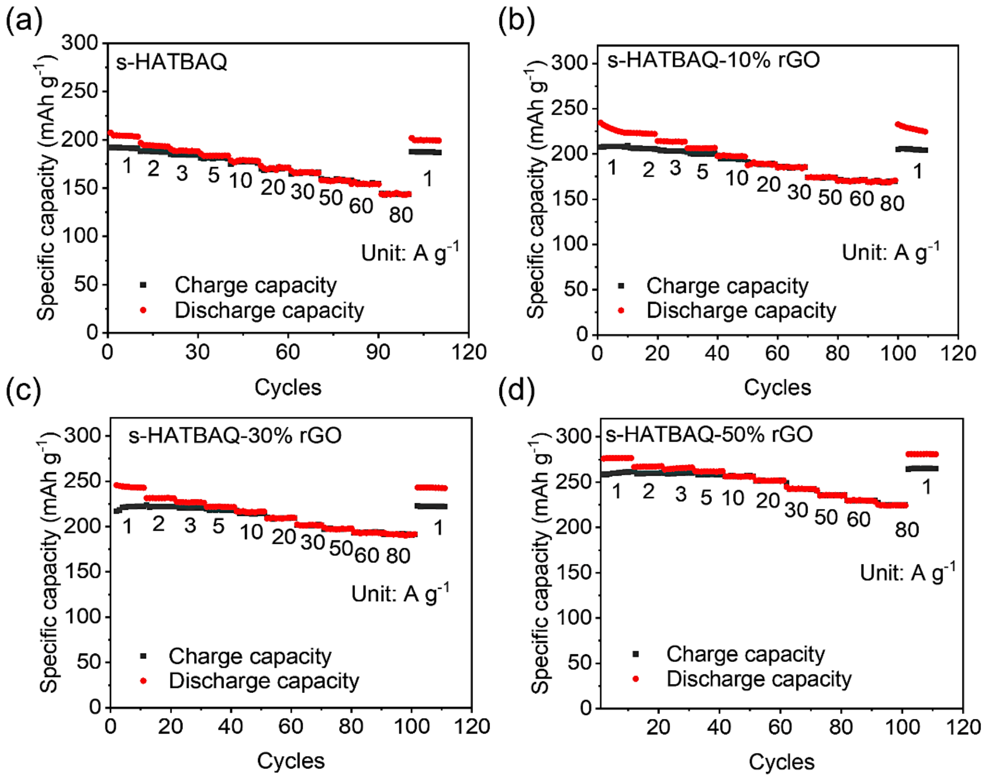


Figure S21. Rate performance of (a) s-HATBAQ, (b) s-HATBAQ-10% rGO, (c) s-HATBAQ-30% rGO, and (d) s-HATBAQ-50% rGO.

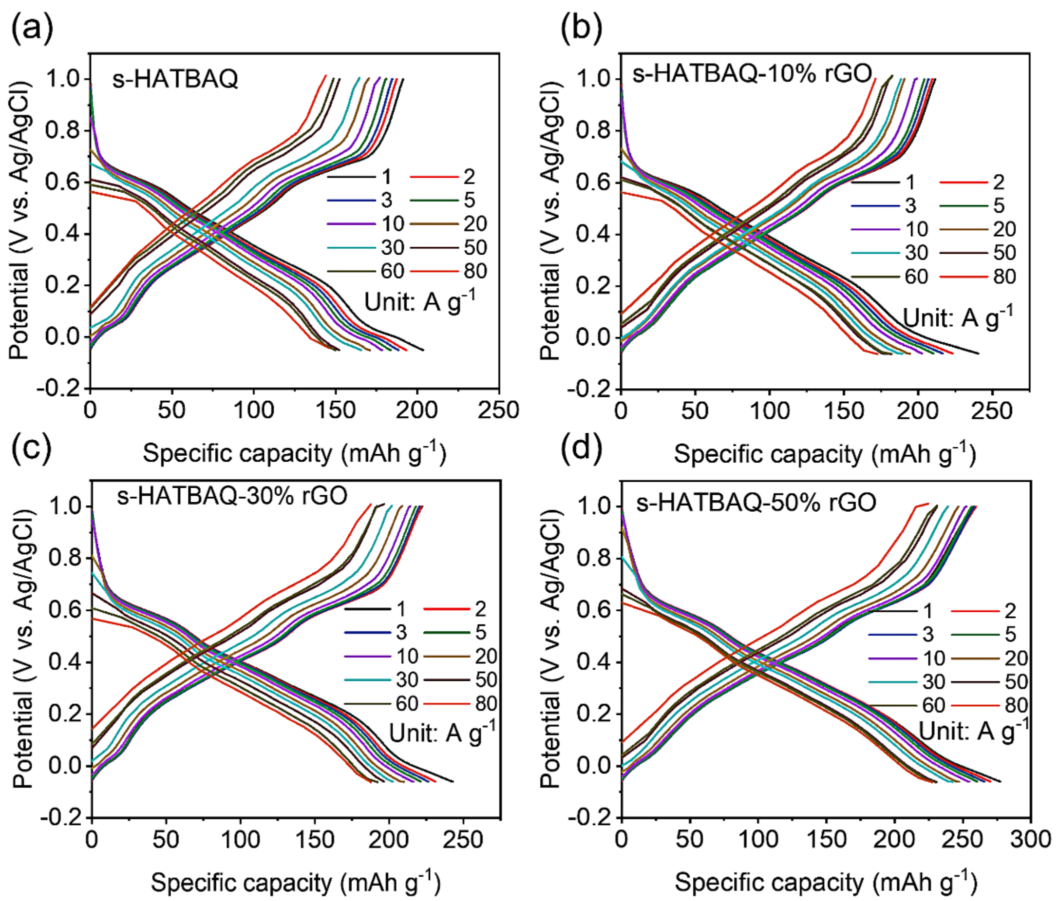


Figure S22. GCD profiles of (a) s-HATBAQ, (b) s-HATBAQ-10% rGO, (c) s-HATBAQ-30% rGO, and (d) s-HATBAQ-50% rGO.

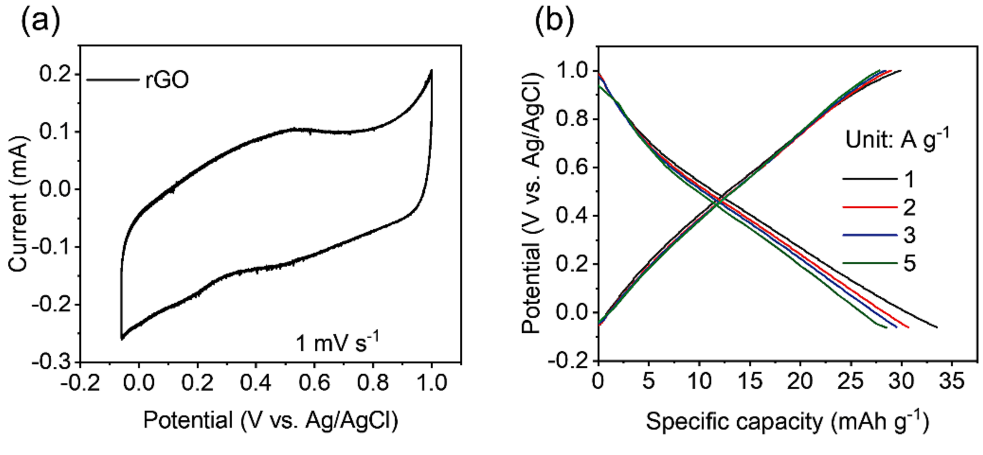


Figure S23. (a) CV curve and (b) GCD profile of rGO.

To explore the advantages of the in-situ growth method, s-HATBAQ/50% rGO composites were prepared by physically mixing the s-HATBAQ and rGO with a weight ratio of 1:1. **Figure S24** shows electrochemical properties of s-HATBAQ/50% rGO. When the physically mixed s-HATBAQ/50% rGO was used as the active material, although s-HATBAQ/50% rGO exhibits similar CV curve profile to s-HATBAQ-50 % rGO (**Figure S24a**), the discharge capacity is only 140.3 mAh $g^{-1}$ at 80 A $g^{-1}$, which is much lower than that of in-situ grown s-HATBAQ-50% rGO composites (231 mAh $g^{-1}$ at 80 A $g^{-1}$) (**Figure S24b**). Additionally, s-HATBAQ/50% rGO shows a capacity retention of 84 % after 5000 cycles (20 A $g^{-1}$), also lower than s-HATBAQ-50% rGO (91.7 %) (**Figure S24c**). Those results demonstrate that in-situ growth method is more advantageous than simple physical mixture, which can be ascribed to sufficient contact between s-HATBAQ and rGO.

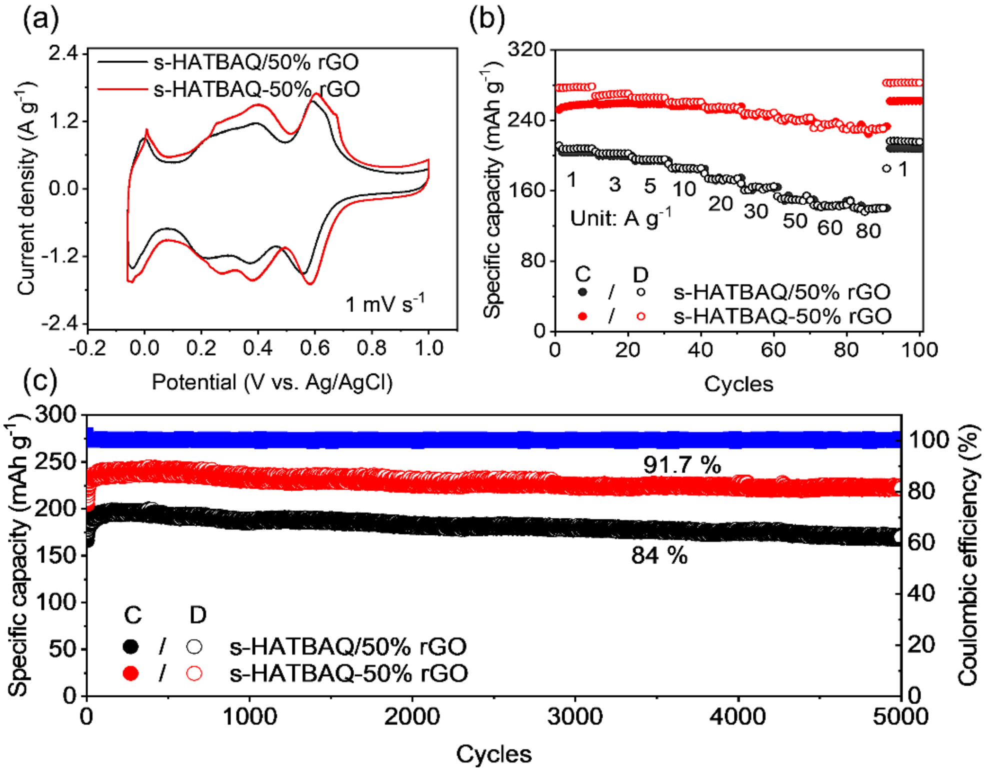


Figure S24. (a) CV curve, (b) Rate performance, and (c) Long cycling stability of s-HATBAQ/50% rGO and s-HATBAQ-50% rGO.

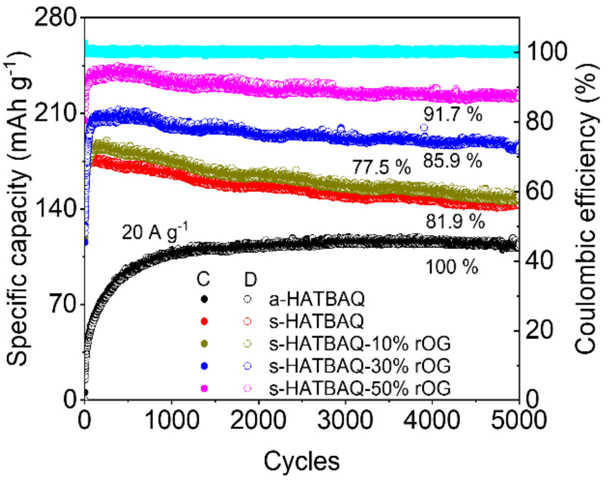


Figure S25. Long cycling stability of a-HATBAQ, s-HATBAQ and s-HATBAQ-rGOs.

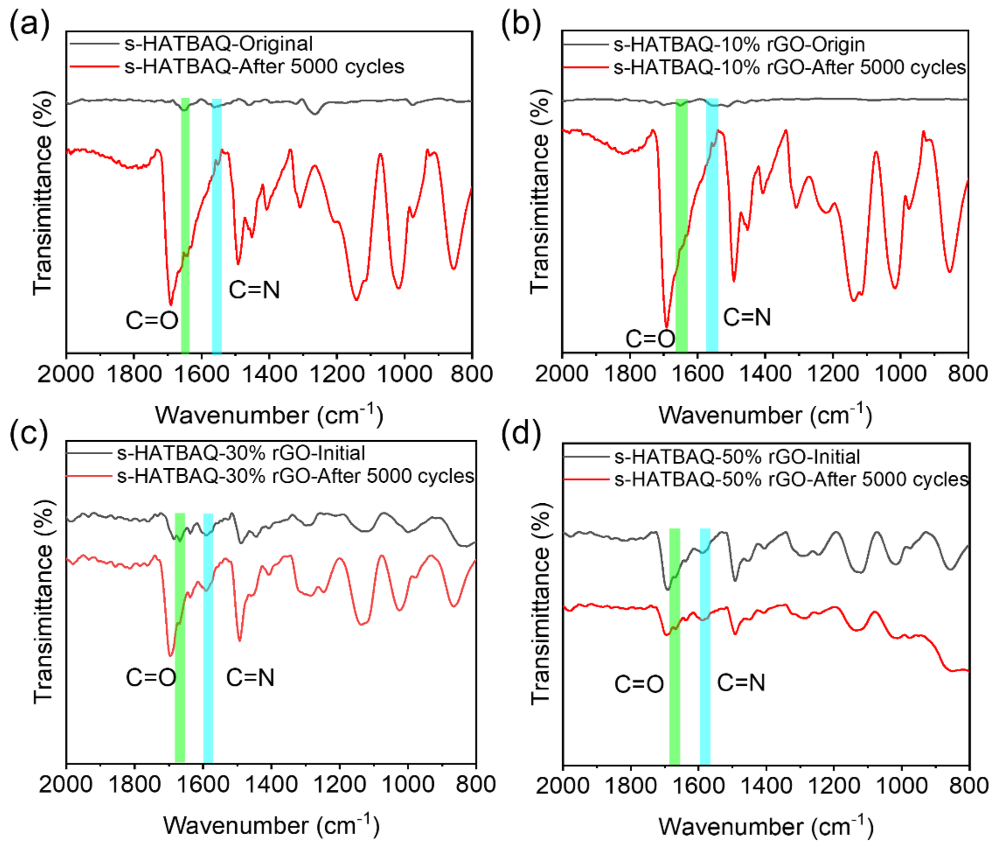


Figure S26. FT-IR spectra of (a) s-HATBAQ, (b) s-HATBAQ-10% rGO, (c) s-HATBAQ-30% rGO, and (d) s-HATBAQ-50% rGO in the initial state and after 5000 cycles at 20 A $g^{-1}$.

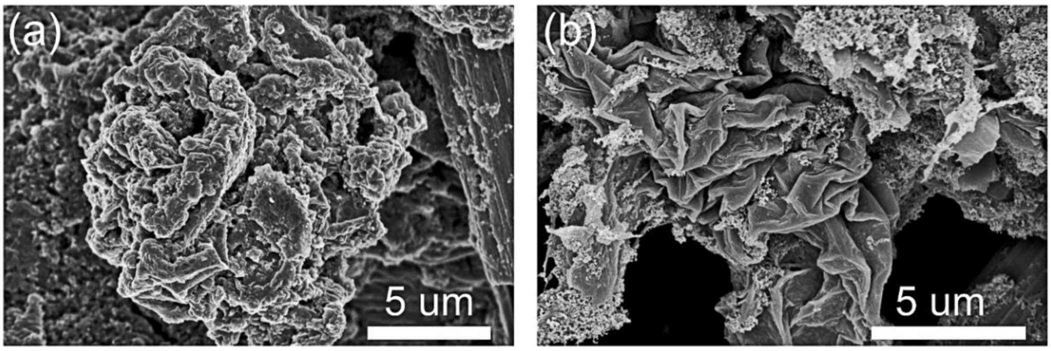


Figure S27. SEM images of (a) s-HATBAQ-30% rGO and (b) s-HATBAQ-50% rGO after 5000 cycles at 20 A $g^{-1}$.

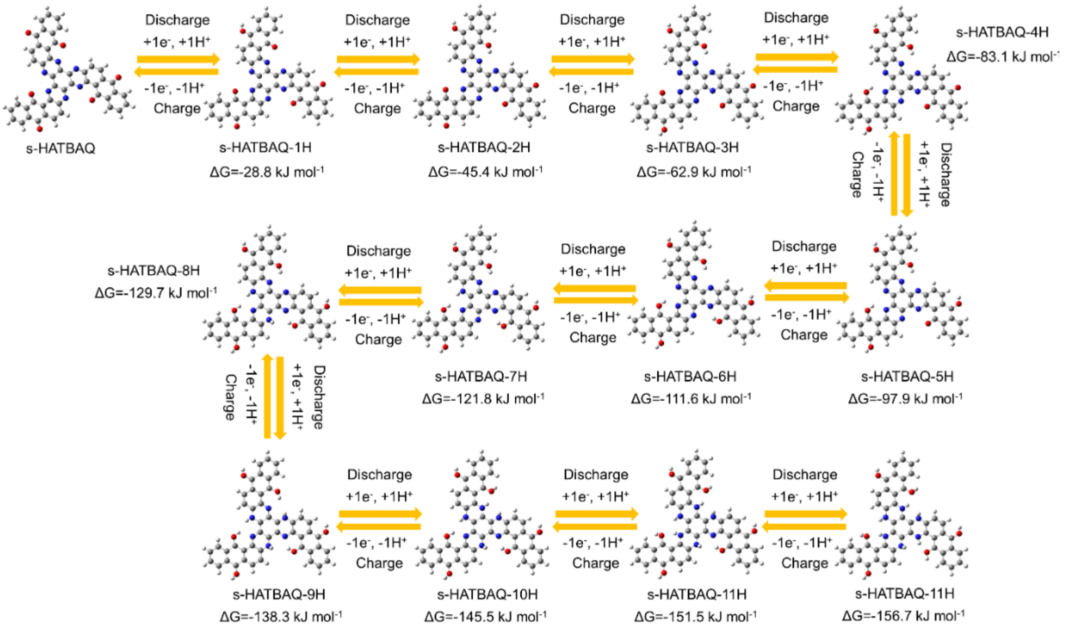


Figure S28. The charge storage mechanism for s-HATBAQ.

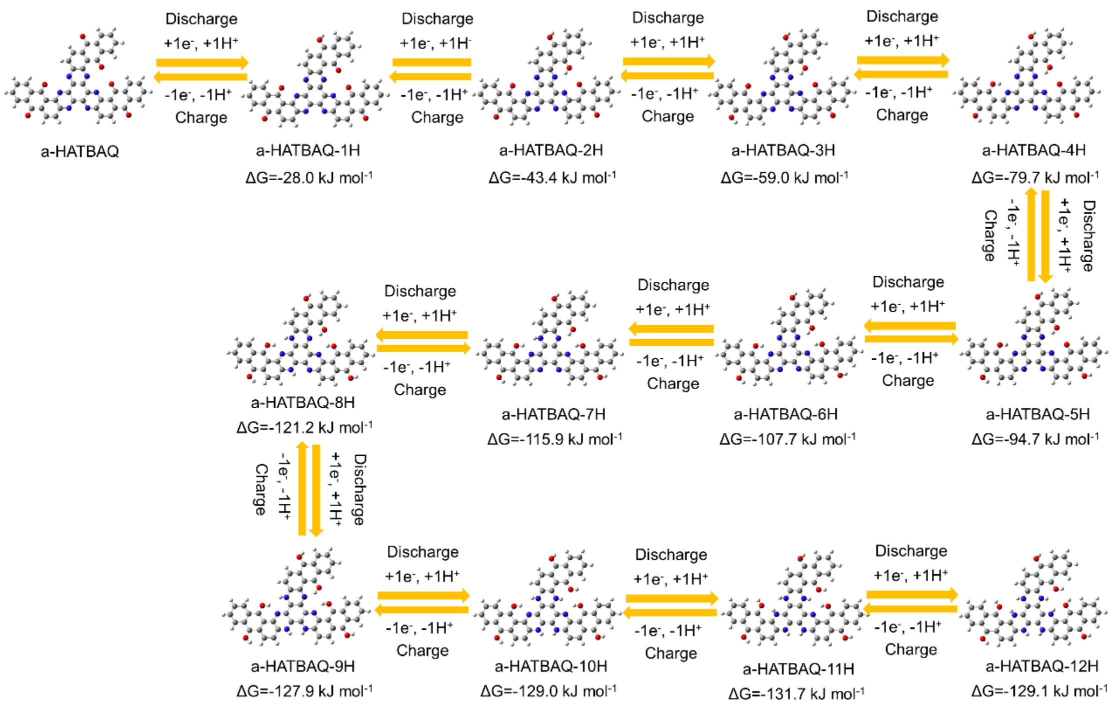


Figure S29. The charge storage mechanism for a-HATBAQ.

As shown in **Figure S30a**, commercial CFs exhibit super-hydrophobic features with a contact angle (CA) of 145.8 °, and the water drops are almost completely immersed in CF-KOH (**Figure S30b, Video S1**), indicating a drastic transition from super-hydrophobicity to super-hydrophilicity after the KOH treatment.

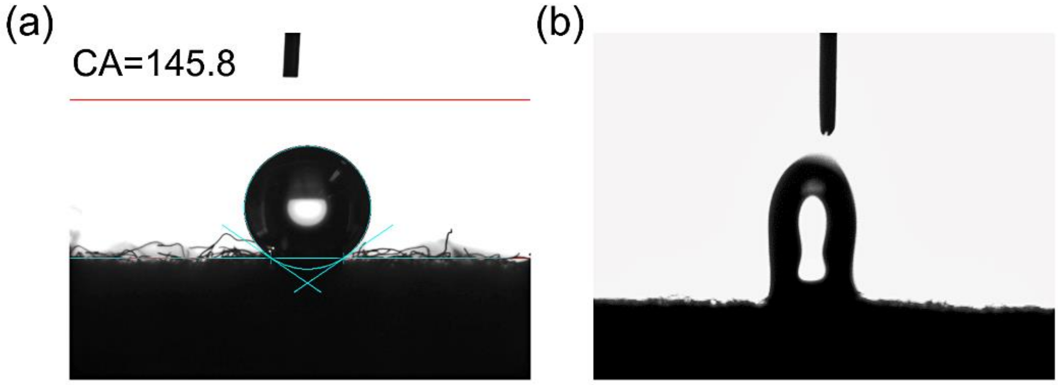


Figure S30. CA values of (a) commercial CF and (b) CF-KOH.

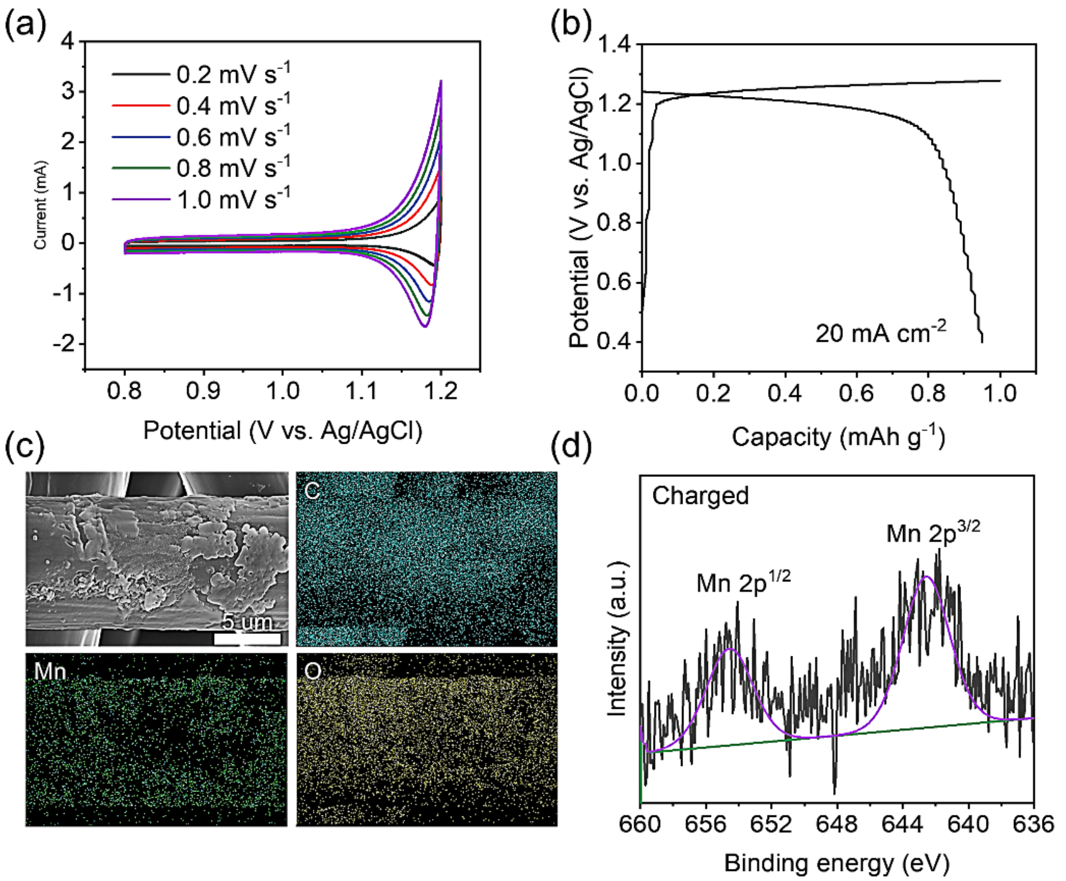


Figure S31. (a) The CV curves of $MnO_2$@CF-KOH in half cells, (b) Charge-discharge curve of $MnO_2$@CF-KOH at 20 mA $cm^{-2}$, (c) SEM images and EDS-elemental mapping images with 15 mAh $MnO_2$ electrodeposition for CF-KOH-electrode Scale bar: 5 μm, and (d) XPS spectra of Mn element on CF-KOH-electrode at charged state at 5 mA $cm^{-2}$.

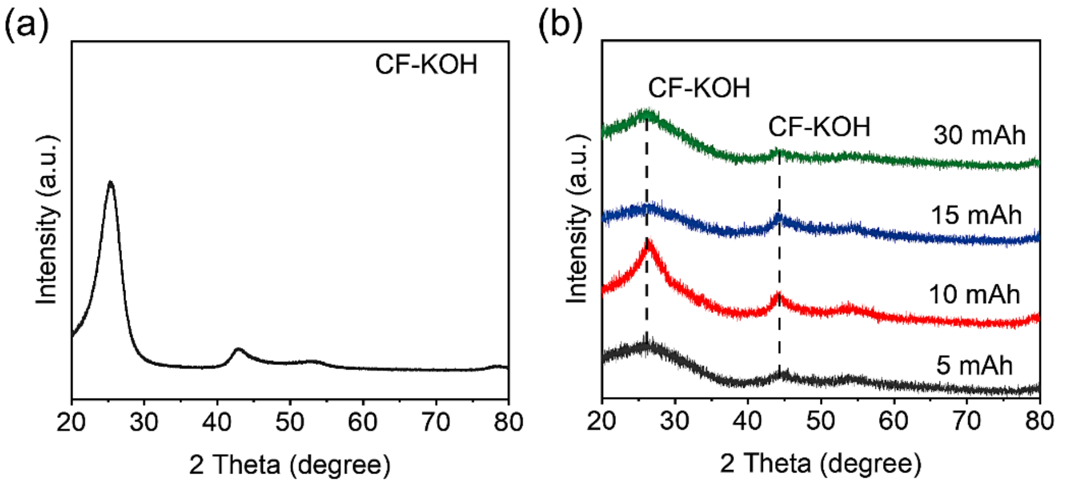


Figure S32. XRD patterns of (a) CF-KOH, and (b) $MnO_2$ with different capacities of 5, 10, 15, and 30 mAh.

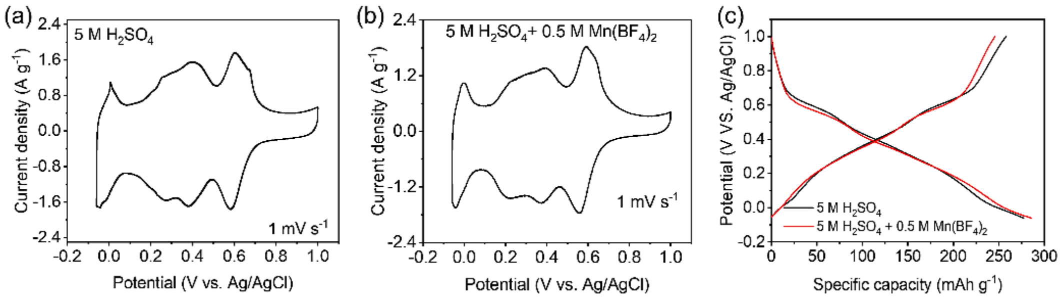


Figure S33. CV curves of (a) s-HATBAQ-50% rGO in 5 M $H_2SO_4$ and (b) s-HATBAQ-50% rGO in 5 M $H_2SO_4$+0.5 M $Mn(BF_4)_2$, and (c) GCD curves of s-HATBAQ-50% rGO at two kinds of electrolyte.

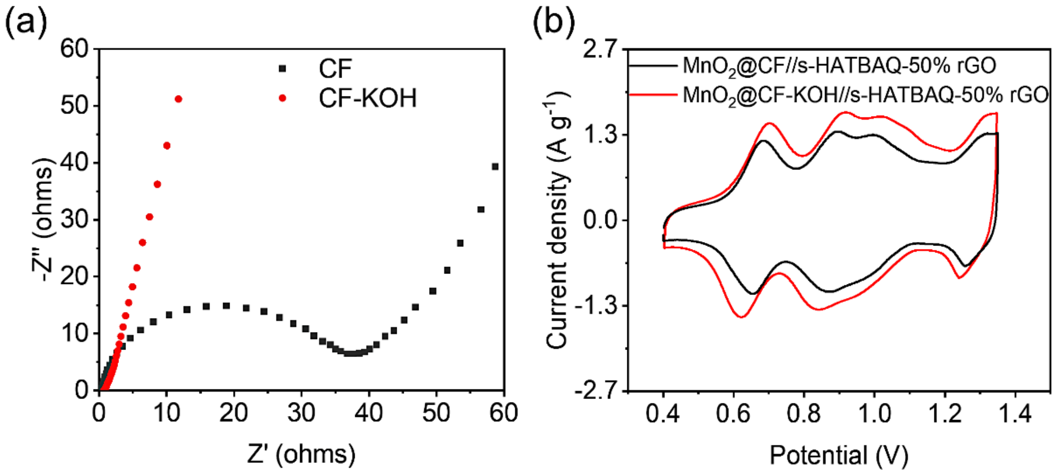


Figure S34. (a) EIS values of CF and CF-KOH, and (b) CV curves of $MnO_2$@CF//s-HATBAQ-50% rGO and $MnO_2$@CF-KOH//s-HATBAQ-50% rGO at 1 mV $s^{-1}$.

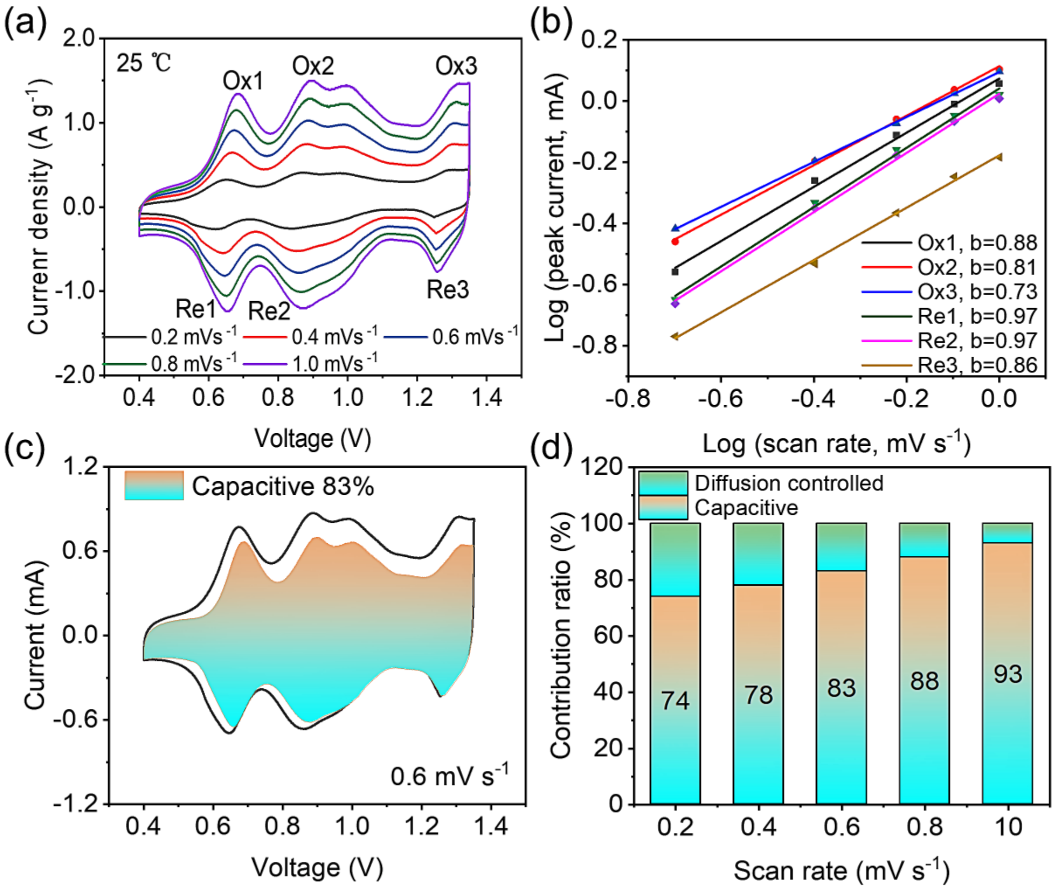


Figure S35. Electrochemical performance of full battery at 25 ℃. (a) CV curves at different scan rates, (b) The corresponding plots of log (i) versus log (v) at each redox peak, (c) Capacitive behaviors and intercalation reaction contributions at 0.6 mV $s^{-1}$, and (d) Contribution ratio of the pseudocapacitance at various scan rates.

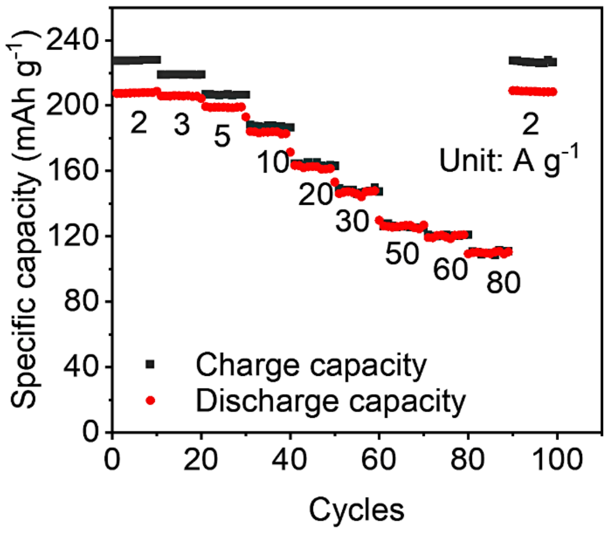


Figure S36. Rate performance of full battery at 25 ℃.

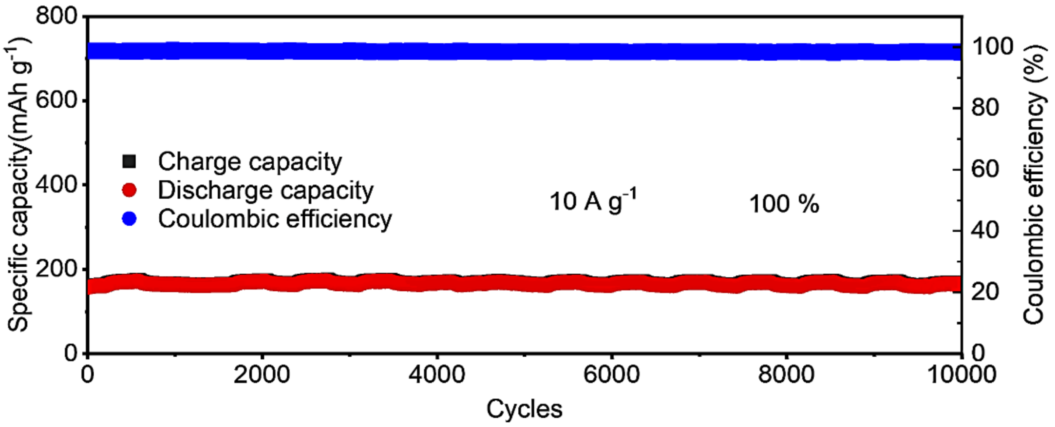


Figure S37. Long cycling performance of full battery 10 A g$^{-1}$ at 25 ℃.

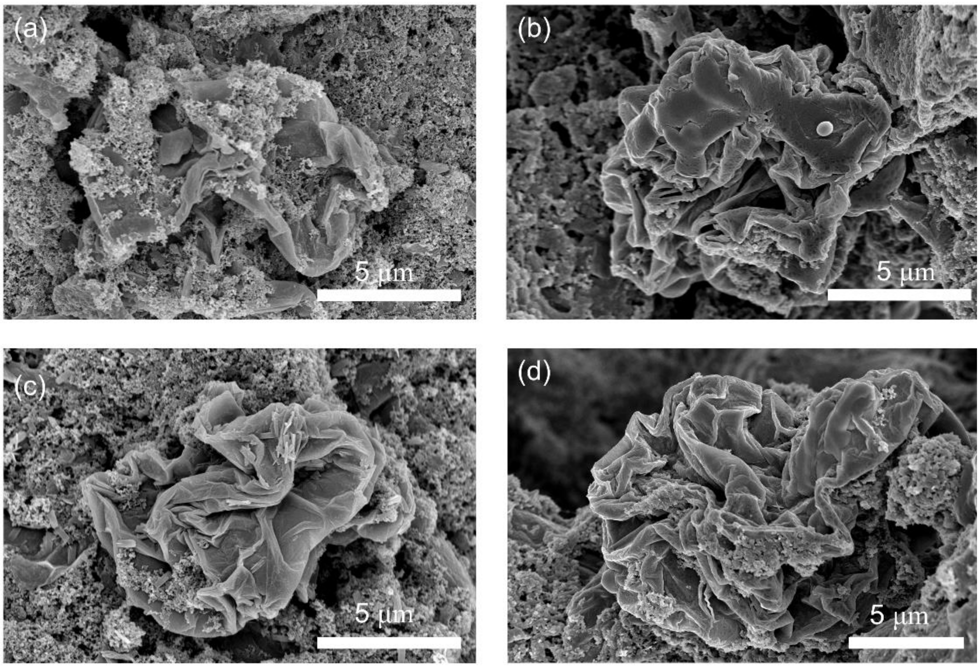


Figure S38. SEM images of (a) Initial state, (b) full battery after 10000 cycles at 10 A g$^{-1}$, (c) full battery after 23000 cycles at 20 A g$^{-1}$, and (d) full battery after 25000 cycles at 5 A g$^{-1}$.

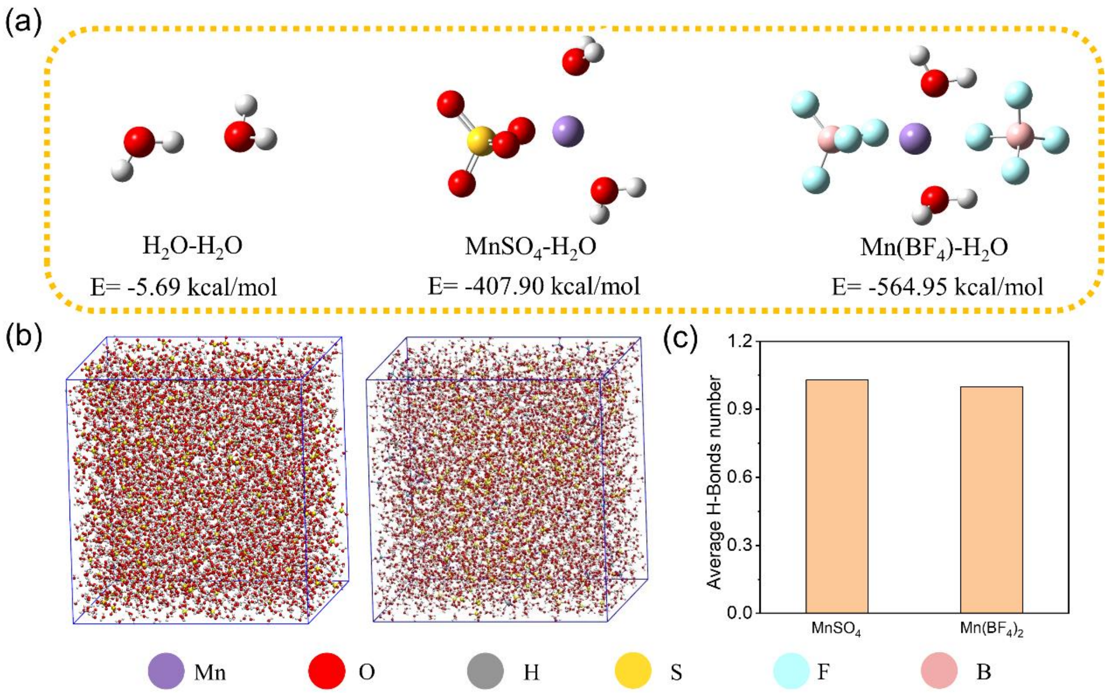


Figure S39. Structure and anti-freezing mechanism of electrolyte. (a) The binding energy between $H_2O$, $MnSO_4$, and $Mn(BF_4)_2$ obtained by DFT simulation, (b) MD simulation snapshots of saturated $MnSO_4$-$H_2SO_4$ electrolyte and saturated $Mn(BF_4)_2$-$H_2SO_4$ electrolyte, and (c) The average number of H-bonds formed between water molecules obtained by MD simulation.

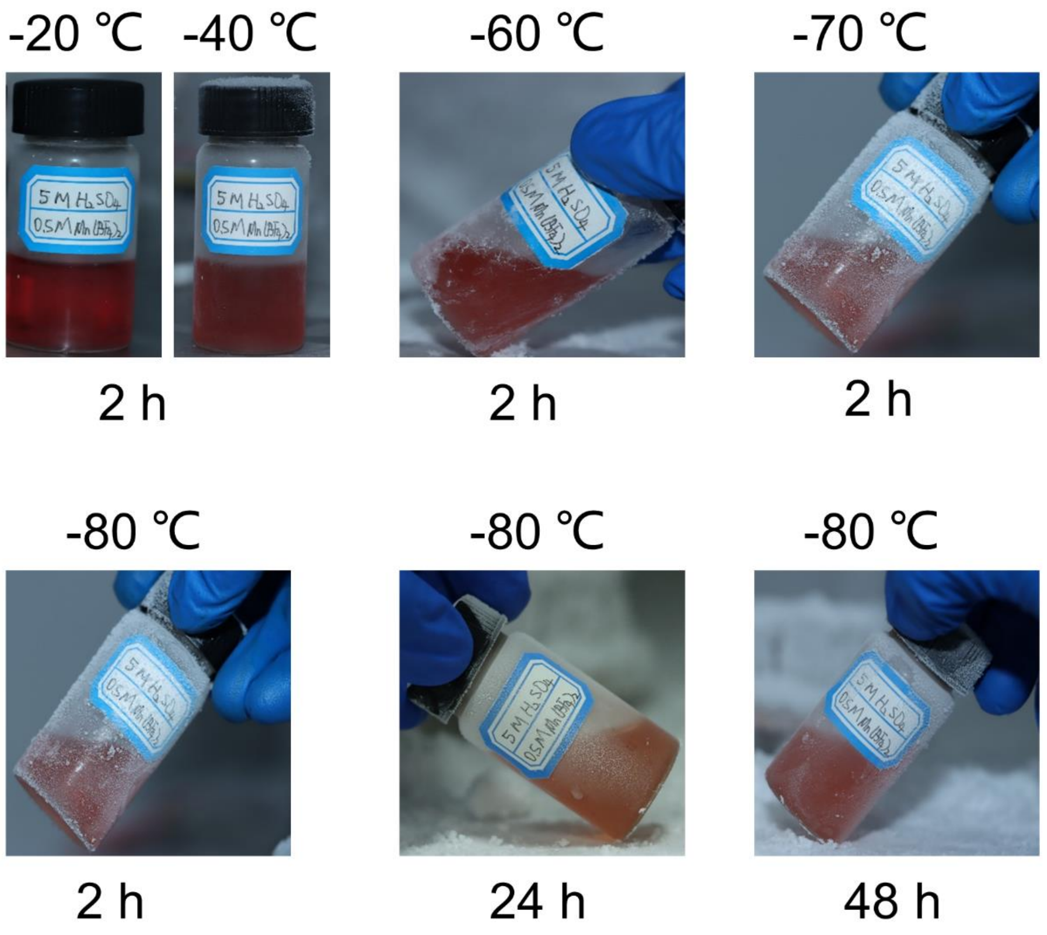


Figure S40. The visual images of electrolytes at different temperatures.

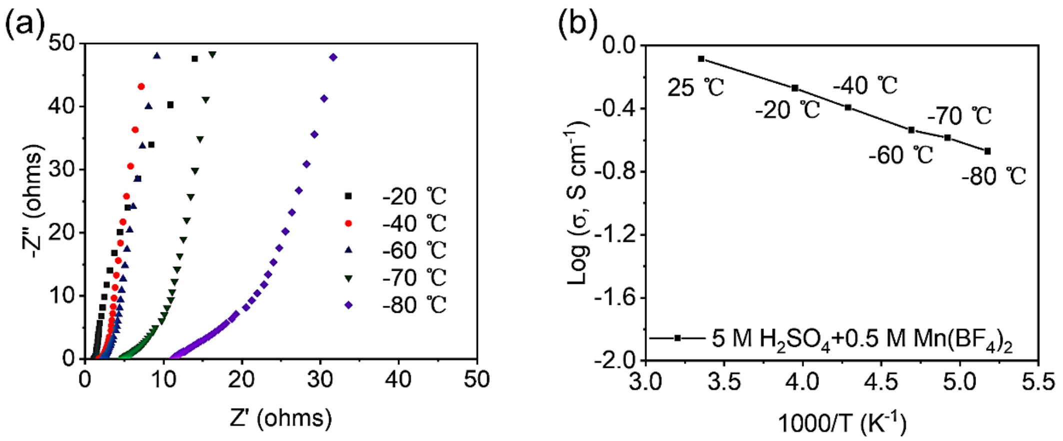


Figure S41. (a) EIS values of full battery at 5 M $H_2SO_4$ + 0.5 M $Mn(BF_4)_2$ electrolyte under different temperatures and (b) Temperature-dependent ionic conductivity investigation for the acid electrolyte containing $Mn^{2+}$.

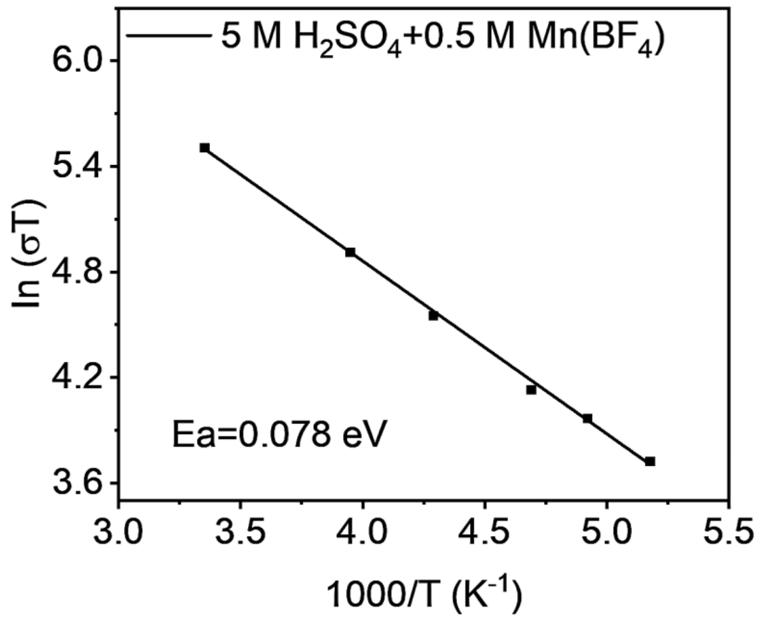


Figure S42. The electric conductance activation energy of 5 M $H_2SO_4$ + 0.5 M $Mn(BF_4)_2$ electrolyte at diff erent temperatures

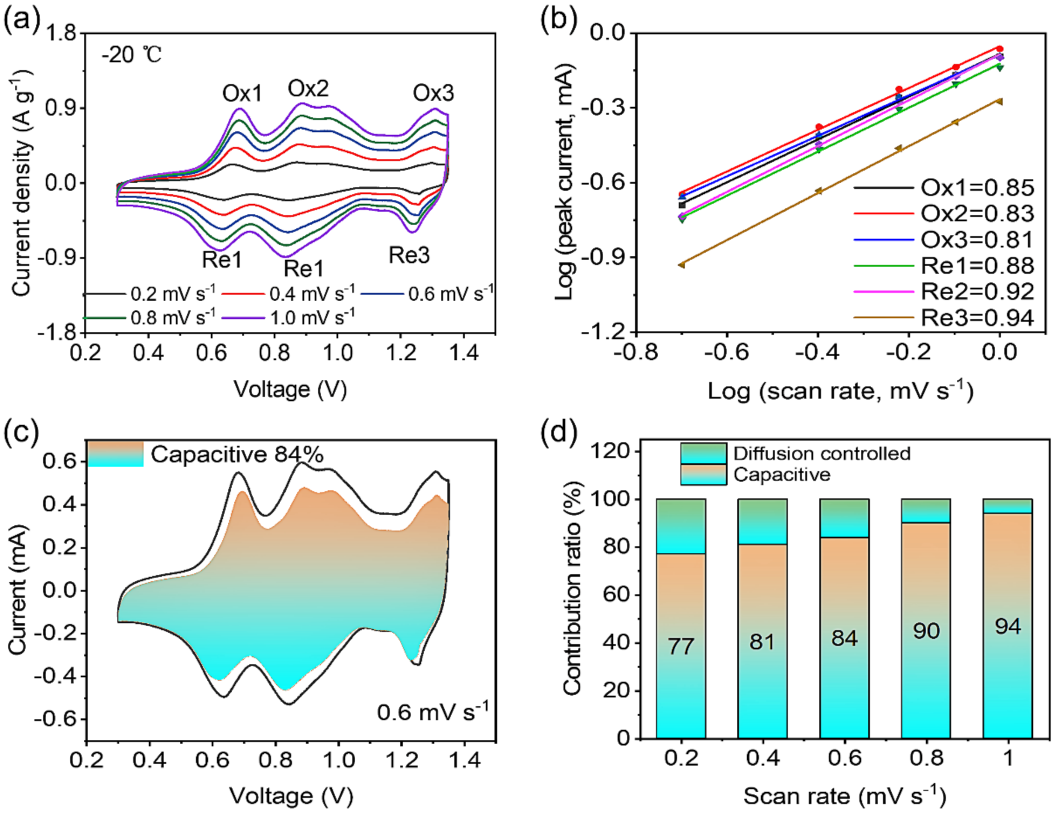


Figure S43. Electrochemical performance of full battery at -20 ℃. (a) CV curves at different scan rates, (b) The corresponding plots of log (i) versus log (v) at each redox peak, (c) Capacitive behaviors and intercalation reaction contributions at 0.6 mV $s^{-1}$, and (d) Contribution ratio of the pseudocapacitance at various scan rates.

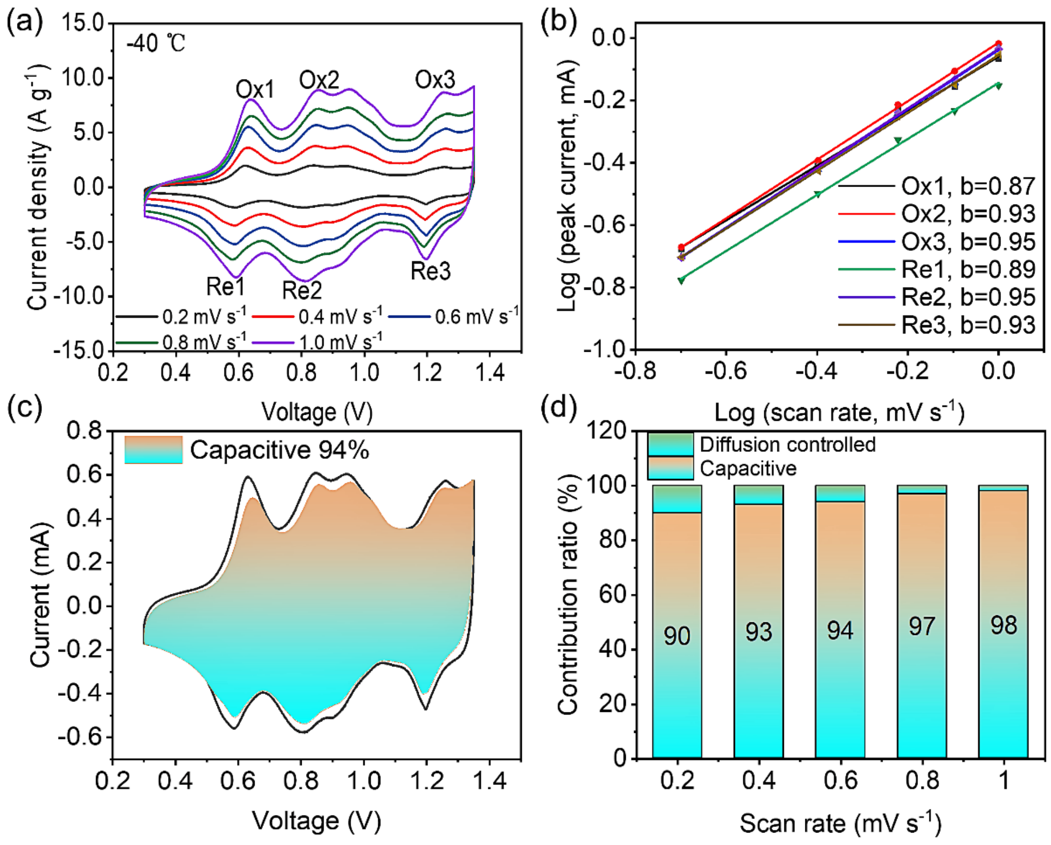


Figure S44. Electrochemical performance of full battery at -40 ℃. (a) CV curves at different scan rates, (b) The corresponding plots of log (i) versus log (v) at each redox peak, (c) Capacitive behaviors and intercalation reaction contributions at 0.6 mV $s^{-1}$, and (d) Contribution ratio of the pseudocapacitance at various scan rates.

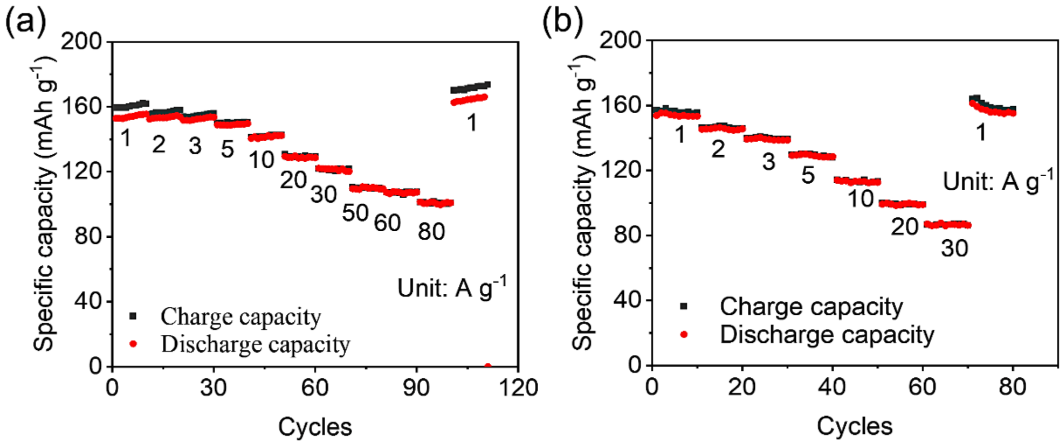


Figure S45. Rate performance of full battery at (a) -20 ℃ and (b) -40 ℃.

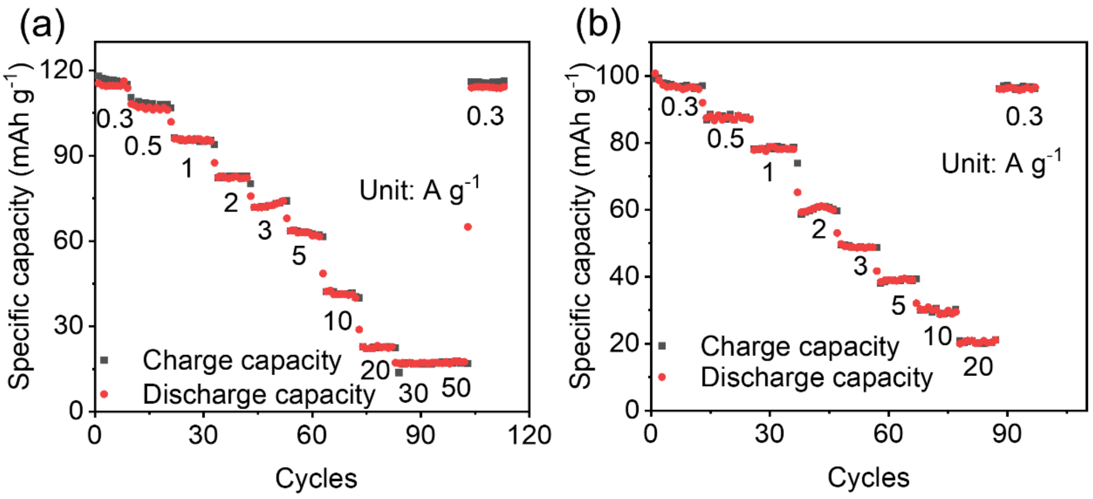


Figure S46. Rate performance of full battery at (a) -60 ℃ and (b) -70 ℃

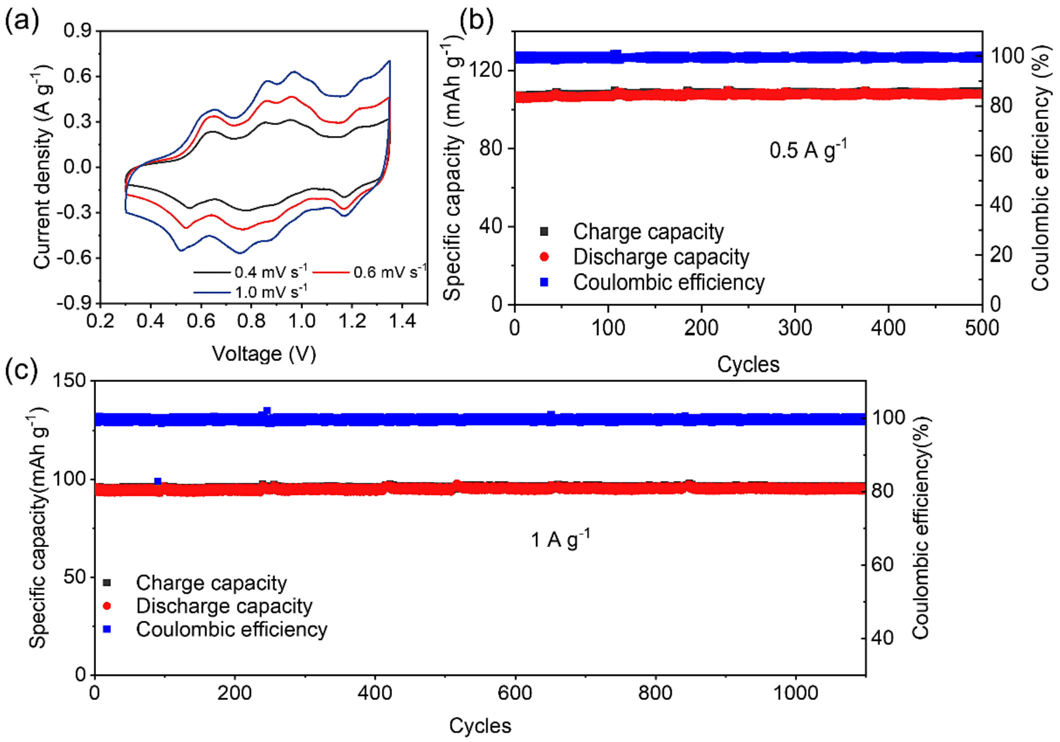


Figure S47. Electrochemical performance of full battery at -60 ℃. (a) CV curves at different scan rates and long cycling stability at (c) 0.5 A $g^{-1}$ and (d) 1 A $g^{-1}$.

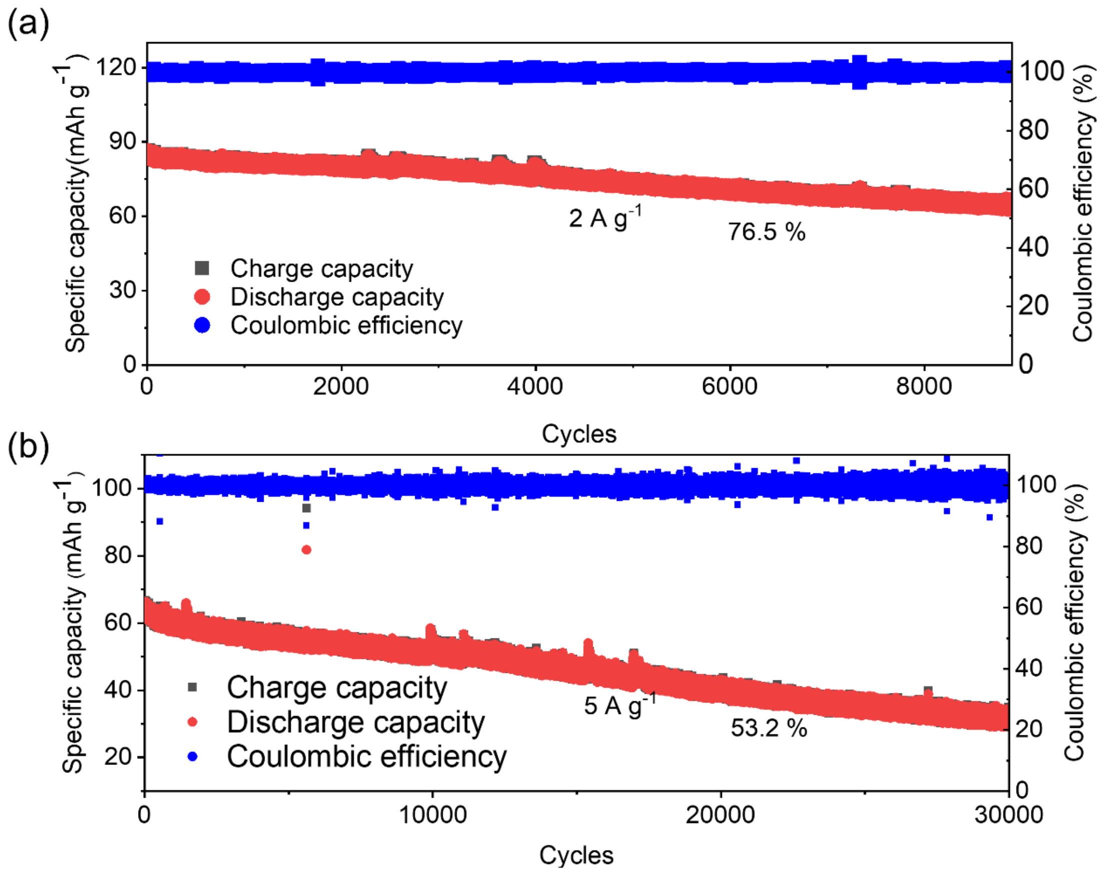


Figure S48. Long cycling stability of full batteries at -60 ℃: (a) 2 A $g^{-1}$, and (b) 5 A $g^{-1}$.

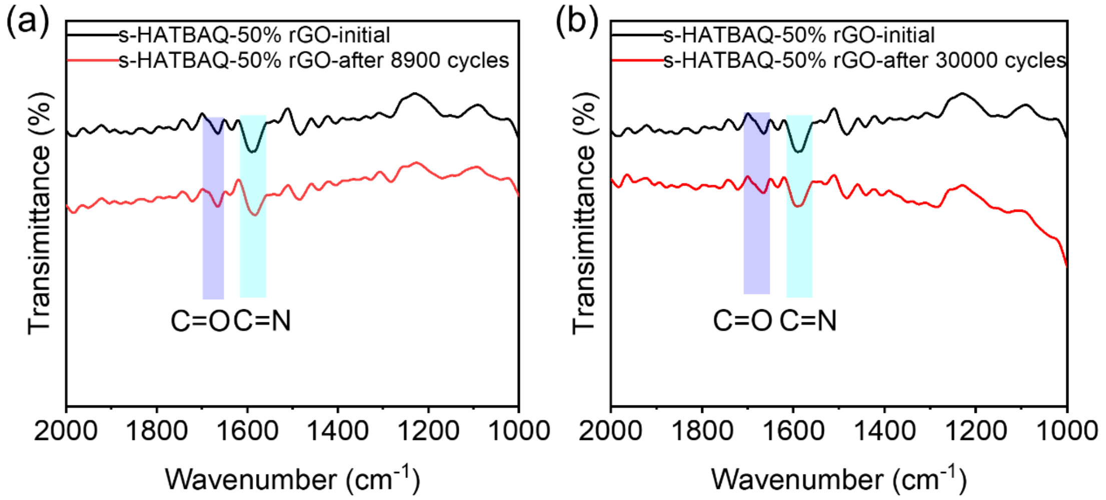


Figure S49. FT-IR spectra of full battery at -60 ℃: (a) at initial state and after 8900 cycles at 2 A $g^{-1}$, and (b) at initial state and after 30000 cycles at 5 A $g^{-1}$.

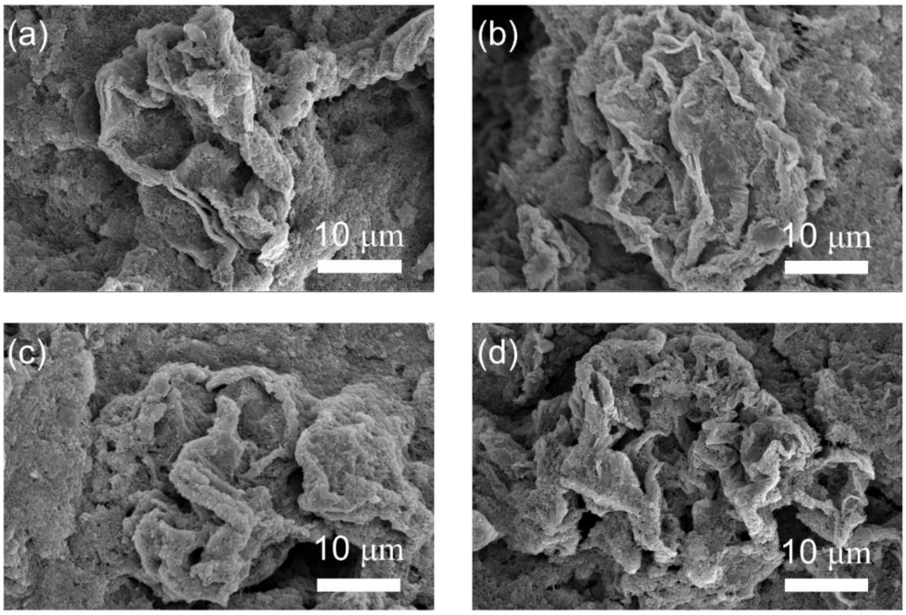


Figure S50. SEM images of s-HATBAQ-50 % rGO at -60 ℃: (a) at initial state, (b) after 8900 cycles at 2 A $g^{-1}$, (c) at initial state, and (d) after 30000 cycles at 5 A $g^{-1}$.

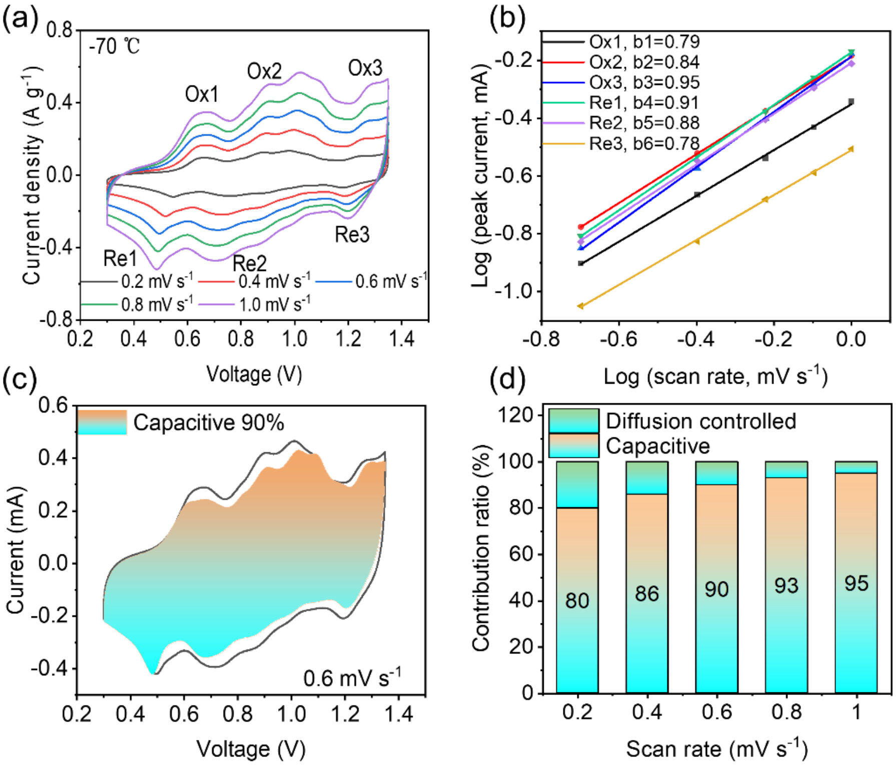


Figure S51. Electrochemical performance of full battery at -70 ℃. (a) CV curves at different scan rates, (b) The corresponding plots of log (i) versus log (v) at each redox peak, (c) Capacitive behaviors and intercalation reaction contributions at 0.6 mV $s^{-1,}$ and (d) Contribution ratio of the pseudocapacitance at various scan rates.

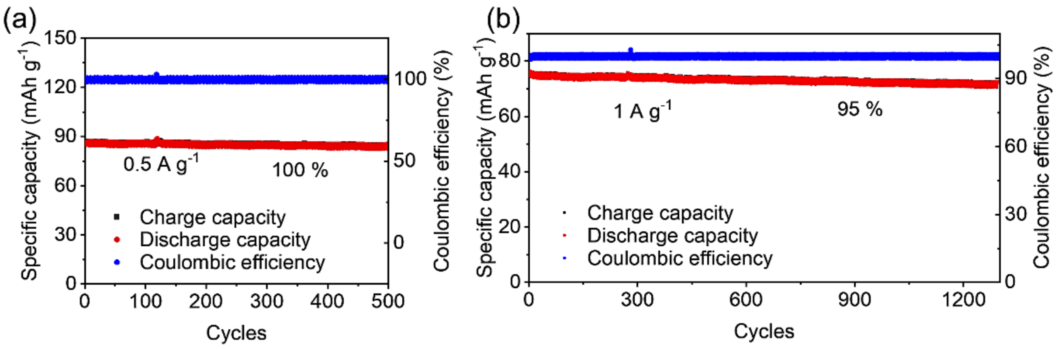


Figure S52. Long cycling stability of full battery at different densities (a) 0.5 A $g^{-1}$ and (b) 1 A $g^{-1}$ at -70 ℃.

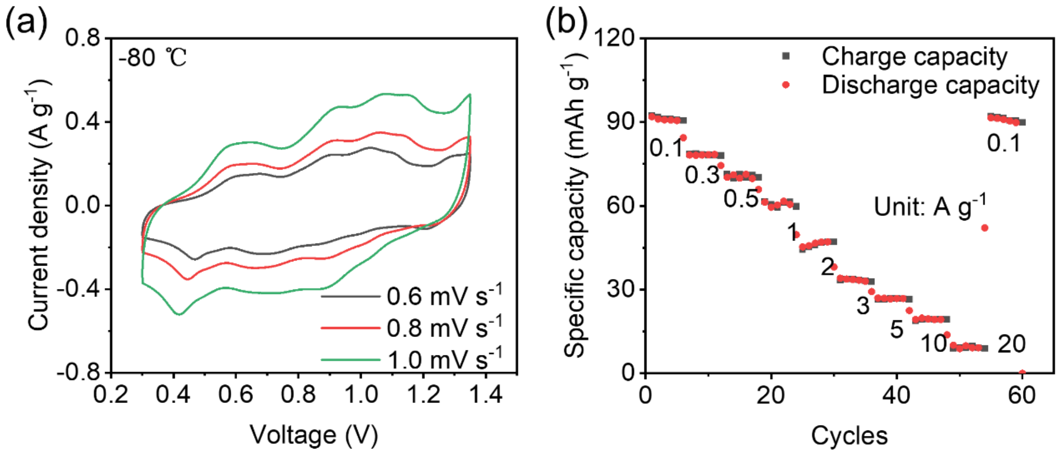


Figure S53. (a) CV curves and (b) rate performance of full battery at -80 ℃.

**Table S1** shows the electrical conductivity of s-HATBAQ, s-HATBAQ-10% rGO, s-HATBAQ-30% rGO, s-HATBAQ-50% rGO, and GO tested under six pressures (2 MPa, 4 MPa, 6 MPa, 8 MPa, 10 MPa, and 12 MPa). At the same pressure, such as 10 MPa, s-HATBAQ-50% rGO shows the highest electrical conductivity (1.21 S $m^{-1}$), followed by s-HATBAQ-30% rGO (1.08 $E^{-1}$ S $m^{-1}$), GO (5.05 $E^{-3}$ S $m^{-1}$), s-HATBAQ-10% rGO (8.19 $E^{-4}$ S $m^{-1}$), and s-HATBAQ (3.65 $E^{-13}$ S $m^{-1}$). Particularly, s-HATBAQ-30% rGO and s-HATBAQ-50% rGO exhibit higher electrical conductivity than GO, further indicating that GO is reduced to rGO during the chemical synthesis of the composites. Additionally, it can be clearly seen that the left-most reduction peak of s-HATBAQ-50% rGO moves to low potential compared with that of HATBAQ, which can be ascribed to the overlapping effect of the hydrogen evolution capacity of reduced graphene oxide at low potential (**Figure S23a**).

Table S1. The electronic conductivity of obtained samples.

| Sample | Test content | Test methods | Result | | | | | | Units |
|---|---|---|---|---|---|---|---|---|---|
| | | | 2 MPa | 4 MPa | 6 Mpa | 8 MPa | 10 MPa | 12 MPa | |
| s-HATBAQ | Electrical conductivity | Quadrupole probe | $9.76E^{-10}$ | $1.47E^{-09}$ | $1.75E^{-09}$ | $2.02E^{-09}$ | $3.65E^{-09}$ | $4.81E^{-09}$ | S/m |
| s-HATBAQ-10% rGO | Electrical conductivity | Quadrupole probe | $2.14E^{-04}$ | $4.00E^{-04}$ | $5.41E^{-04}$ | $8.37E^{-04}$ | $8.19E^{-04}$ | $8.62E^{-04}$ | S/m |
| s-HATBAQ-30% rGO | Electrical conductivity | Quadrupole probe | $1.33E^{-02}$ | $3.42E^{-02}$ | $5.98E^{-02}$ | $8.50E^{-02}$ | $1.08E^{-01}$ | $1.30E^{-01}$ | S/m |
| s-HATBAQ-50% rGO | Electrical conductivity | Quadrupole probe | $1.65E^{-01}$ | $4.11E^{-01}$ | $6.96E^{-01}$ | $9.61E^{-01}$ | $1.21E^{+00}$ | $1.41E^{+00}$ | S/m |
| GO | Electrical conductivity | Quadrupole probe | $3.57E^{-03}$ | $3.77E^{-03}$ | $4.37E^{-03}$ | $4.73E^{-03}$ | $5.05E^{-03}$ | $5.16E^{-03}$ | S/m |

**Capacity Contribution of rGO in the s-HATBAQ-rGO composites**

The specific capacities of the **s-HATBAQ-rGO** composites were calculated based on the mass of the **s-HATBAQ** in the composite. However, both **s-HATBAQ** and **rGO** contribute to the overall capacity of the electrode, so the total specific capacity of the PT-COFX composites was calculated using Equation S3 **[14]**:

$$C_{s\text{-}HATBAQ\text{-}rGOs} = \frac{a\times m\times C_{s\text{-}HATBAQ} + b\times m\times C_{rGO}}{a\times m} \quad (3)$$

Where $C_{s\text{-}HATBAQ\text{-}rGOs}$ and $C_{s\text{-}HATBAQ}$ are the specific capacities of the s-HATBAQ-rGOs and s-HATBAQ, respectively, in the composite electrodes. $C_{rGO}$ is the specific capacity of pure rGO (34 mAh $g^{-1}$) without s-HATBAQ, and m is the mass of the s-HATBAQ-rGOs, a and b are the contents of s-HATBAQ and rGO in the s-HATBAQ-rGOs, such that a+b=1. Therefore, the capacity contribution of rGO in the s-HATBAQ-rGOs is $C_{s\text{-}HATBAQ\text{-}rGOs} - C_{s\text{-}HATBAQ} = (b\times C_{rGO})/a$

Table S2. Capacity contribution of rGO in the s-HATBAQ-rGOs.

| | Calculation of capacity contribution of rGO in the s-HATBAQ-rGOs / mAh $g^{-1}$ |
|---|---|
| s-HATBAQ-10% rGOs | 0.1×34/0.9 = 4 |
| s-HATBAQ-30% rGOs | 0.3×34/0.7 = 15 |
| s-HATBAQ-50% rGOs | 0.5×34/0.5 = 34 |

Table S3. The electronic conductivity of s-HATBAQ and a-HATBAQ

| | 2MPa | 4MPa | 6MPa | 8MPa | Unit |
|---|---|---|---|---|---|
| s-HATBAQ | $9.76E^{-10}$ | $1.47E^{-09}$ | $1.75E^{-09}$ | $2.02E^{-09}$ | s/m |
| a-HATBAQ | $1.84E^{-09}$ | $2.52E^{-09}$ | $3.27E^{-09}$ | $3.63E^{-09}$ | s/m |

Table S4. Gibbs free energies of the protonated s-HATBAQ molecules in each step.

| Samples | G $_{\text{Gibbs free energies}}$ (kJ $mol^{-1}$) |
|---|---|
| s-HATBAQ | 0 |
| s-HATBAQ-1H | -28.8 |
| s-HATBAQ-2H | -45.4 |
| s-HATBAQ-3H | -62.9 |
| s-HATBAQ-4H | -83.1 |
| s-HATBAQ-5H | -97.9 |
| s-HATBAQ-6H | -111.6 |
| s-HATBAQ-7H | -121.8 |
| s-HATBAQ-8H | -129.7 |
| s-HATBAQ-9H | -138.3 |
| s-HATBAQ-10H | -145.5 |
| s-HATBAQ-11H | -151.5 |
| s-HATBAQ-12H | -156.7 |

Table S5. Gibbs free energies of the protonated a-HATBAQ molecules in each step.

| Samples | G $_{\text{Gibbs free energies}}$ (kJ $mol^{-1}$) |
|---|---|
| a-HATBAQ | 0 |
| a-HATBAQ-1H | -28 |
| a-HATBAQ-2H | -43.4 |
| a-HATBAQ-3H | -59 |
| a-HATBAQ-4H | -79.7 |
| a-HATBAQ-5H | -94.7 |
| a-HATBAQ-6H | -107.7 |
| a-HATBAQ-7H | -115.9 |
| a-HATBAQ-8H | -121.2 |
| a-HATBAQ-9H | -127.9 |
| a-HATBAQ-10H | -129 |
| a-HATBAQ-11H | -131.7 |
| a-HATBAQ-12H | -129.1 |

As shown in **Table S6**, the O mass ratio of CF-KOH is almost 10 times that of CF, indicating that abundant oxygen-containing groups are responsible for the excellent hydrophilic features of CF-KOH

Table S6. Elemental analysis of CF and CF-KOH.

| Samples | C (%) | H (%) | S (%) | O (%) |
|---|---|---|---|---|
| CF | 94.15 | 0.1 | 0 | 0.11 |
| CF-KOH | 84.83 | 0.13 | 0 | 1.99 |

Comparison of performance with literature

Table S7. Rate performance and cycling performance comparison of $MnO_2$@CF-KOH//HATBAQ-50% rGO battery with other materials-based proton battery reported in the literature.

| Samples | | Discharge capacity (mAh/g) /Current density (A/g) | Cycling performance (Current density) | Ref. |
|---|---|---|---|---|
| PR/PO | 2 M $ZnSO_4$ | 147 (0.1 A $g^{-1}$); 90 (2 A $g^{-1}$) | 94 % / After 500 cycles (1A $g^{-1}$) | [15] |
| $MnO_2$@GF//ALO (r.m.) | 2 M $H_2BF_4$+2 M $MnBF_4$ | 145.5 (1 A $g^{-1}$) | <90 % / After 300 cycles (5 A $g^{-1}$) | [16] |
| $MnO_2$@GF//ALO (-60 ℃) | 2 M $H_2BF_4$+2 M $MnBF_4$ | 101.8 (1 A $g^{-1}$); 100 (1.5 A $g^{-1}$) | 100 % / After 500 cycles (1 A $g^{-1}$) | [16] |
| $MnO_2$@GF//TMBQ-rGO (r.m.) | 0.5 M H2SO4 + 1 M MnSO4 | 148 (32.6 A $g^{-1}$) | 77 % / After 4000 cycles (1.63 A $g^{-1}$) | [17] |
| PCHL-rGO//Pb(r.m.) | 5 M $H_2SO_4$ | 208 (0.2 A $g^{-1}$); 84 (100 A $g^{-1}$) | 67% / After 3000 cycles (10 A $g^{-1}$) | [18] |

| | | | | |
|---|---|---|---|---|
| PCHL-rGO//Pb(-70 ℃) | 5 M $H_2SO_4$ | 87 (0.1 A $g^{-1}$) | 97 % / After 500 cycles (0.5A $g^{-1}$) | [18] |
| $MnO_2$@GF//MoO3 (r.m.) | 2 M $H_2SO_4$+2M $MnSO_4$ | 209 (1 A $g^{-1}$); 103 (60 A $g^{-1}$) | 81 % / After 300 cycles (4 A $g^{-1}$) | [19] |
| $MnO_2$@GF//$MoO_3$ (-70 ℃) | 2 M $H_2SO_4$+2M $MnSO_4$ | 171.8 (0.1 A $g^{-1}$); <125 (0.5 A $g^{-1}$) | 100 % / After 100 cycles (0.2 A $g^{-1}$) | [19] |
| $MnO_2$@GF//PTO (r.m.) | 2 M $H_2SO_4$+2M $MnSO_4$ | 150 (0.2 A $g^{-1}$); <125 (20 A $g^{-1}$) | 80 %/After 5000 cycles (1 A $g^{-1}$) | [20] |
| $MnO_2$@GF//PTO (-70 ℃) | 2 M $H_2SO_4$+2M $MnSO_4$ | 110 (0.2 A $g^{-1}$); 89 (1 A $g^{-1}$) | 99 % / After 100 cycles (0.4 A $g^{-1}$) | [20] |
| $MnO_2$@CC//$MoO_3$@$TiO_2$ (r.m.) | 1 M $H_2SO_4$+1M $MnSO_4$ | 200.8 (1 A $g^{-1}$); 116.3 (20 A $g^{-1}$) | 80 % / After 500 cycles (10 A $g^{-1}$) | [21] |
| PTC//PUQ (r.m.) | 0.5 M $H_2SO_4$ | 78.1 (0.5 A $g^{-1}$); 50.8 (25 A $g^{-1}$) | 80 % / After 1000 cycles (2 A $g^{-1}$) | [22] |
| PNAO//PNAO (r.m.) | 4 M $H_2SO_4$ | 85 (0.6 A $g^{-1}$) | | [23] |
| PNAO//PNAO (-70 ℃) | 4 M $H_2SO_4$ | 60.4 (0.6 A $g^{-1}$) | 100 % / After 200 cycles (1.2 A $g^{-1}$) | [23] |
| H-TBA//$MoO_3$ (r.m.) | 9.5 M $H_3PO_4$ | 44 (5 A $g^{-1}$); 34 (50 A $g^{-1}$) | | [24] |
| H-TBA//$MoO_3$ (-78 ℃) | 9.5 M $H_3PO_4$ | 28 (0.025 A $g^{-1}$) | 100 % / After 450 cycles (0.26 A $g^{-1}$) | [24] |

| | | | | |
|---|---|---|---|---|
| Pin//PDQPZ | 1M $H_2SO_4$ | 133.3 (0.1 A $g^{-1}$) | | [25] |
| CuFePBA @MXene//PDPZ@ MXene | 2 M $H_2SO_4$ | 65.1 (1 A $g^{-1}$) | 98.2%/After 10000 cycles | [26] |
| $MnO_2$@GF//DTT | 2 M $H_2SO_4$+2M $MnSO_4$ | 208 (0.05 A $g^{-1}$); 90 (10 A $g^{-1}$) | | [27] |
| $MnO_2$@GF//DTT (-60 ℃) | 2 M $H_2SO_4$+2M $MnSO_4$ | 108 (0.05 A $g^{-1}$); 77 (1 A $g^{-1}$) | | [27] |
| $MnO_2$@GF//DTT (-70 ℃) | 2 M $H_2SO_4$+2M $MnSO_4$ | 98 (0.05 A $g^{-1}$); < 60 (1 A $g^{-1}$) | | [27] |
| $MnO_2$@GF-KOH// HATBAQ-50% rGO (r.m.) | 5 M $H_2SO_4$+0.5M $Mn(BF_4)_2$ | 210 (2 A $g^{-1}$); 184 (10 A $g^{-1}$); 111 (80 A $g^{-1}$) | 96 % / After 25000 cycles (5 A $g^{-1}$); 100 % / After 10000 cycles (10 A $g^{-1}$); 100 % / After 23000 cycles (20 A $g^{-1}$) | This Work |
| $MnO_2$@GF-KOH// HATBAQ-50% rGO (-60 ℃) | 5 M $H_2SO_4$+0.5M $Mn(BF_4)_2$ | 114.2 (0.3 A $g^{-1}$); 95.8 (1 A $g^{-1}$); 82.3 (3 A $g^{-1}$); 63 (5 A $g^{-1}$) | 100 % / After 1100 cycles (1 A $g^{-1}$); 100 % / After 500 cycles (0.5 A $g^{-1}$); | This Work |
| $MnO_2$@GF-KOH// HATBAQ-50% rGO (-70 ℃) | 5 M $H_2SO_4$+0.5M $Mn(BF_4)_2$ | 88.1 (0.5 A $g^{-1}$); 78.1 (1A $g^{-1}$); 61.1 (2 A $g^{-1}$) | 95 % / After 1200 cycles (1 A $g^{-1}$); 100 % / After 500 cycles (0.5 A $g^{-1}$); | This Work |
| $MnO_2$@CF-KOH// HATBAQ-50% rGO (-80 ℃) | 5 M $H_2SO_4$+0.5M $Mn(BF_4)_2$ | 91 (0.1 A $g^{-1}$); 78 (0.3 A $g^{-1}$); 70 (0.5 A $g^{-1}$) | 92 % / After 1000 cycles (0.5 A $g^{-1}$); | This Work |

Table S8. Energy density and power density comparison of $MnO_2$@CF-KOH//HATBAQ-50% rGO battery with other materials-based proton battery reported in the literature.

| Samples | Energy density | Power density | Operating Voltage | Ref. |
|---|---|---|---|---|
| $MnO_2$@GF//PTO (r.m.) | 132.6 Wh $kg^{-1}$ (total mass of anode and consumed cathode) | 30.8 kW k $g^{-1}$ | 0.3-1.3 | [20] |
| $MnO_2$@GF//ALO (-60 ℃) | 132.6 Wh $kg^{-1}$ (based on the anode mass) | 1650 W $kg^{-1}$ | 0.8-1.4 | [16] |
| $MnO_2$@GF//TMBQ-rGO (r.m.) | 166.4 Wh $kg^{-1}$ (based on the total active mass of both the cathode and anode) | / | / | [17] |
| $MnO_2$@GF//MoO 3 (r.m.) | 177.4 Wh $kg^{-1}$ (total mass of anode and consumed cathode) | 66.6 kW k $g^{-1}$ | 0.8-1.6 | [19] |
| $MnO_2$@GF//$MoO_3$ @TiO2 (r.m.) | 252.9 Wh $kg^{-1}$ (total mass of anode and consumed cathode) | 18.3 kW k $g^{-1}$ | 0.8-1.6 | [21] |
| PTC//PUQ (r.m.) | 56.2 Wh $kg^{-1}$ (the total mass of the anode and cathode) | 360 W k $g^{-1}$ | 0-1.2 | [22] |
| H-TBA//$MoO_3$ (-78 ℃) | 24 Wh $kg^{-1}$ (active mass of both electrodes at the current rate of 25 mA $g^{-1}$) | / | 0-1.6 | [24] |

| | | | | |
|---|---|---|---|---|
| $MnO_2$@GF-KOH//HATBAQ-50% rGO (r.m.) | 178 Wh $kg^{-1}$ total mass of anode , 2 A $g^{-1}$) | 1487 W $kg^{-1}$ | 0.4-1.35 | This work |
| $MnO_2$@GF-KOH//HATBAQ-50% rGO (-60 ℃) | 74.4 Wh $kg^{-1}$ (total mass of anode and consumed cathode, 1 A $g^{-1}$) | 547.1 W k $g^{-1}$ | 0.3-1.35 | This work |
| $MnO_2$@GF-KOH//HATBAQ-50% rGO (-70 ℃) | 47.0 Wh kg-1 (total mass of anode and consumed cathode, 1 A $g^{-1}$) | 537.7 W k $g^{-1}$ | 0.3-1.35 | This work |
| $MnO_2$@GF-KOH//HATBAQ-50% rGO (-80 ℃) | 36.6 Wh kg-1 (total mass of anode and consumed cathode, 1 A $g^{-1}$) | 310 W k $g^{-1}$ | 0.3-1.35 | This work |

**Supplemental Notes**

**Note S1. Discussions of $MnO_2/Mn^{2+}$ electrolysis**

Nevertheless, electrochemical deposition of $MnO_2$ on a conductive substrate such as carbon fiber via manganese solution ($MnCl_2$, $MnSO_4$) to fabricate a cathode material for proton batteries has been investigated previously. According to the published literatures **[21-24]**, $MnO_2/Mn^{2+}$ electrolysis was discussed as follow. The electrolytic reaction begins with the adsorption of $Mn^{2+}$ ions in $Mn(BF_4)_2$ solution onto electrode surfaces through diffusion [S1], where the $Mn^{2+}$ are electrochemically oxidized to the soluble intermediates of $Mn^{3+}$ [S2]. In 5 M $H_2SO_4$+0.5 M $Mn(BF_4)_2$ electrolyte, $Mn^{3+}$ appear to sustain an extended life span and prevail in disproportionation reaction by $Mn^{3+}$-$Mn^{3+}$ collision and the following hydrolysis to form $MnO_2$ [S3-S5].

$$Mn^{2+}_{electrolyte} \rightleftarrows Mn^{2+}_{ads} \qquad [S1]$$

$$Mn^{2+}_{ads} \longrightarrow Mn^{3+}_{ads} \text{ (electrochemical oxidation)} \qquad [S2]$$

In 5 M $H_2SO_4$+0.5 M $Mn(BF_4)_2$

$$Mn^{3+}_{ads} \rightleftarrows Mn^{3+}_{electrolyte} \qquad [S3]$$

$$Mn^{3+} + Mn^{3+} \longrightarrow Mn^{2+} + Mn^{4+} \text{ (disproportionation)} \qquad [S4]$$

$$Mn^{4+} + 2\,H_2O \longrightarrow MnO_2 + 4\,H^+ \qquad [S5]$$

## Reference


1. Zhao Y, Truhlar DG. The M06 suite of density functionals for main group thermochemistry, thermochemical kinetics, noncovalent interactions, excited states, and transition elements: two new functionals and systematic testing of four M06-class functionals and 12 other functionals. *Theor Chem Acc* 2008; **120**: 215-41.
2. Petersson GA, Bennett A, Tensfeldt TG *et al.* A complete basis set model chemistry .1. The total energies of closed-shell atoms and hydrides of the 1st-row elements. *J Chem Phys* 1988; **89**: 2193-218.
3. Petersson GA, Allaham MA. A complete basis set model chemistry .2. Open-shell systems and the total energies of the 1st-row atoms. *J Chem Phys* 1991; **94**: 6081-90.
4. Lu T, Chen QX. Shermo: A general code for calculating molecular thermochemistry properties. *Comput Theor Chem* 2021; **1200**: 113249.
5. Vujovic M, Ragavendran V, Arsic B *et al.* DFT calculations as an efficient tool for prediction of Raman and infra-red spectra and activities of newly synthesized cathinones. *Open Chem* 2020; **18**: 185-95.
6. Humphrey W, Dalke A, Schulten K. VMD: Visual molecular dynamics. *J Mol Graph Model* 1996; **14**: 33-8.
7. Abraham MJ, Murtola T, Schulz R *et al.* GROMACS: High performance molecular simulations through multi-level parallelism from laptops to supercomputers. *SoftwareX* 2015; 1-2, 19-25.
8. William, L., Jorgensen. Erratum: Monte carlo simulation of n‐butane in water. Conformational evidence for the hydrophobic effect. *J Chem Phys* 1982; **77**: 5757-65.
9. Hristov IH, Paddison SJ, Paul R. Molecular modeling of proton transport in the short-side-chain perfluorosulfonic acid ionomer. *J Phys Chem B* 2008; **112**: 2937-49.
10. Li PF, Merz KM. Taking into account the ion-induced dipole interaction in the nonbonded model of ions. *J Chem Theory Comput*. 2014; **10**: 289-97.
11. Williams CD, Carbone P. A classical force field for tetrahedral oxyanions developed using hydration properties: The examples of pertechnetate (TcO) and sulfate (SO). *J Chem Phys* 2015; **143**: 174502.
12. Rappé AK, Casewit CJ, Colwell K *et al.* UFF, a full periodic table force field for molecular mechanics and molecular dynamics simulations. *J Am Chem Soc* 1992; **114**: 10024-35.
13. Wang J, Wolf RM, Caldwell JW *et al.* Development and testing of a general amber force field. *Journal of computational chemistry* 2004; **25**: 1157-74.
14. Gao H, Neale AR, Zhu Q *et al.* A pyrene-4,5,9,10-tetraone-based covalent organic framework delivers high specific capacity as a Li-ion positive electrode. *J Am Chem Soc* 2022; **144**: 9434-42.
15. Tie ZW, Deng SZ, Cao HM *et al.* A symmetric all-organic proton battery in mild electrolyte. *Angew Chem Int Edit* 2022; **61**: e202115180.
16. Sun TJ, Du HH, Zheng SB *et al.* High power and energy density aqueous proton battery operated at -90 degrees C. *Adv Funct Mater* 2021; **31**: 2010127.
17. Yang XR, Ni YX, Lu Y *et al.* Designing quinone-based anodes with rapid kinetics for rechargeable proton batteries. *Angew Chem Int Edit* 2022; **61**: e202209642.

18. Yue F, Tie ZW, Deng SZ *et al.* An ultralow temperature aqueous battery with proton chemistry. *Angew Chem Int Edit* 2021; **60**: 13882-6.
19. Yan L, Huang JH, Guo ZW *et al.* Solid-state proton battery operated at ultralow temperature. *ACS Energy Lett* 2020; **5**: 685-91.
20. Guo ZW, Huang JH, Dong XL *et al.* An organic/inorganic electrode-based hydronium-ion battery. *Nat Commun* 2020; **11**: 959.
21. Wang CG, Zhao SS, Song XX *et al.* Suppressed dissolution and enhanced desolvation in core-shell $MoO_3$@$TiO_2$ nanorods as a high-rate and long-life anode material for proton batteries. *Adv Energy Mater* 2022; **12**: 2200157.
22. Zhu MH, Zhao L, Ran Q *et al.* Bioinspired catechol-grafting PEDOT cathode for an all-polymer aqueous proton battery with high voltage and outstanding rate capacity. *Adv Sci* 2022; **9**: 2103896.
23. Sun TJ, Du HH, Zheng SB *et al.* Bipolar organic polymer for high performance symmetric aqueous proton battery. *Small Methods* 2021; **5**: 2100367.
24. Jiang H, Shin W, Ma L *et al.* A high-rate aqueous proton battery delivering power below-78 degrees C via an unfrozen phosphoric acid. *Adv Energy Mater* 2020; **10**: 2000968.
25. He J, Zhao Y, Yan C *et al.* Highly redox-active polymer with extensive electron delocalization and optimized molecular orbitals for extraordinary proton storage. *Chem Eng J* 2023; **470**: 144204.
26. Shi MJ, Wang RY, Li LY *et al.* Redox-active polymer integrated with MXene for ultra-stable and fast aqueous proton storage. *Adv Funct Mater* 2023; **33**: 2209777.
27. Wang YR, Wang CX, Wang W *et al.* Organic hydronium-ion battery with ultralong life. *ACS Energy Lett* 2023; **8**:1390-6.
28. Guo HC, Wan LY, Tang JQ *et al.* Stable colloid-in-acid electrolytes for long life proton batteries. *Nano Energy* 2022; **102**: 107642.
29. Chao DL, Ye C, Xie FX *et al.* Atomic engineering catalyzed $MnO_2$ electrolysis kinetics for a hybrid aqueous battery with high power and energy density. *Adv Mater* 2020; **32**: 2001894.
30. Xie CX, Li TY, Deng CZ *et al.* A highly reversible neutral zinc/manganese battery for stationary energy storage. *Energ Environ Sci* 2020; **13**: 135-43.
